# Treg-specific deletion of the phosphatase SHP-1 impairs control of inflammation *in vivo*

**DOI:** 10.3389/fimmu.2023.1139326

**Published:** 2023-03-16

**Authors:** QinLei Gu, Kenneth S. Tung, Ulrike M. Lorenz

**Affiliations:** ^1^ Department of Microbiology, Immunology, and Cancer Biology, University of Virginia, Charlottesville, VA, United States; ^2^ Beirne B. Carter Center for Immunology Research, University of Virginia, Charlottesville, VA, United States; ^3^ Department of Pathology, University of Virginia, Charlottesville, VA, United States; ^4^ Department of Pathology and Immunology, Washington University in St. Louis, Saint Louis, MO, United States

**Keywords:** SHP-1, tyrosine phosphatase, regulatory T cells, T cell signaling, inflammatory disease

## Abstract

**Introduction:**

To achieve a healthy and functional immune system, a delicate balance exists between the activation of conventional T cells (Tcon cells) and the suppression by regulatory T cells (Treg). The tyrosine phosphatase SHP-1, a negative regulator of TCR signaling, shapes this ‘activation-suppression’ balance by modulating Tcon cell resistance to Treg-mediated suppression. Treg cells also express SHP-1, but its role in influencing Treg function is still not fully understood.

**Methods:**

We generated a Treg-specific SHP-1 deletion model, *Foxp3^Cre+^ Shp-1^f/f^*, to address how SHP-1 affects Treg function and thereby contributes to T cell homeostasis using a combination of *ex vivo* studies and *in vivo* models of inflammation and autoimmunity.

**Results:**

We show that SHP-1 modulates Treg suppressive function at different levels. First, at the intracellular signaling level in Treg cells, SHP-1 attenuates TCR-dependent Akt phosphorylation, with loss of SHP-1 driving Treg cells towards a glycolysis pathway. At the functional level, SHP-1 expression limits the *in vivo* accumulation of CD44hiCD62Llo T cells within the steady state Tcon populations (both CD8+ as well as CD4+ Tcon). Further, SHP-1-deficient Treg cells are less efficient in suppressing inflammation *in vivo*; mechanistically, this appears to be due to a failure to survive or a defect in migration of SHP-1-deficient Treg cells to peripheral inflammation sites.

**Conclusion:**

Our data identify SHP-1 as an important intracellular mediator for fine-tuning the balance between Treg-mediated suppression and Tcon activation/resistance.

## Introduction

Our immune system relies on multiple levels of controls to balance its response to danger while maintaining self-tolerance. One crucial participant of these control mechanisms are regulatory T cells (Treg) (1), which actively take part in controlling allergy (2), autoimmunity (3), anti-tumor immunity (4), metabolic regulation (5) and tissue repairing (6). Treg cells are mainly recognized for their suppressive function that keep excessive inflammatory responses under control, although emerging data suggest additional roles of Treg cells in non-immune settings during tissue regeneration, such as in stimulating hair follicle (7) and muscle repair (8). Since the initial identification of FOXP3 as a Treg master transcription factor (9–13), the discovery of additional types of regulatory T cells has continued (14). In humans, FOXP3 is also expressed in activated effector conventional T cells (Tcon) without any associated suppressive activities (15–17); however, in mice, Foxp3 is still considered as the exclusive master transcription factor of Treg cells (18). The term ‘regulatory T cells’ can refer to different T cells exhibiting suppressive activities (19), including the most widely recognized CD4+CD25+FOXP3+ suppressive T cells (9, 18), CD4+FOXP3-IL10+ T regulatory type 1 cells (20, 21), and the CD8+FOXP3+ cells (22–24). In this study, we focus primarily on the CD4+ FOXP3+ CD25+ Treg cells, characterized by constitutive expression of nuclear Foxp3 and the surface proteins CD25, CD45RO, GITR, CD122, HLA-DR, CCR4 and CTLA-4 in human (12) and CD25, CD45RB^low^, CTLA-4, and GITR in mice (11). Treg cells can either be derived from the thymus as part of the thymic T cell development or differentiated from naïve conventional CD4+ T cells in the periphery. In addition, of therapeutic relevance, Treg cells can also be artificially induced *ex vivo* (25, 26).

Under physiological conditions, the activation of CD8 and CD4 Tcons and their suppression by Treg cells offset each other, thereby contributing to a balanced immune response and homeostasis. A disturbance of this equilibrium can lead to pathophysiological phenotypes, such as the failure in suppression favoring autoimmune diseases, such as IPEX (27), while a hyper-suppressive environment within the tumor may hinder anti-tumor immunity (28). Although the exact details of Treg-mediated suppression are complex and incompletely understood, several potential suppressive mechanisms have been described: IL-2 competition by Treg cells (29–31); secretion of immunosuppressive cytokines (32–35); generation of other molecules including immunosuppressive miRNA (36, 37), cAMP (38–40) and adenosine (41, 42); modulation of co-stimulatory molecule expression on antigen presenting cells (APC) (43–46); or inducing cell death (47, 48). Although many mechanisms of suppression have been reported over the years, our understanding of the signaling pathways that regulate Treg activity is still relatively limited.

Src homology region 2 domain-containing phosphatase 1 (SHP-1) is a tyrosine phosphatase that is ubiquitously expressed in all hematopoietic cell lineages (49). SHP-1 can localize to the lipid rafts and interact with early components downstream of the TCR signaling (50), such as Lck (51) and Zap-70 (52). Through these interactions, SHP-1 can negatively regulate TCR signaling pathway (53–55), thereby affecting the downstream T cell function including proliferation, cell death, T cell differentiation, cytokine production, and adhesion (56–58). Previous data from our lab demonstrated that the *motheaten* mouse strain, which globally lacks SHP-1 due to a splicing mutation (59), carries Treg cells that are hyper-active both *in vitro* and *in vivo* (60) However, global loss of SHP-1 causes spontaneous inflammation and autoimmunity at a young age (61); this complicates the ability to discern phenotypes that are cell intrinsic and those emerging from the overall inflammatory environment. Moreover, we have observed that SHP-1 can also modulate signaling in Tcon cells which further regulates the susceptibility to Treg-mediated suppression (62), suggesting a critical role for SHP-1 as an immune-balance modifier. In addition, elevated SHP-1 expression has been associated with several types of tumors, such as epithelial ovarian cancers (63) and high-grade breast cancers (64), suggesting SHP-1 as a potential therapeutic target.

In the present study, we analyzed the functional role of SHP-1 in Treg cells to gain a better mechanistic understanding of how SHP-1 coordinates the immune homeostasis/response under physiological and pathophysiological conditions. We generated a Treg-specific SHP-1 deletion model, *Foxp3* ^Cre+^ *Shp-1* ^f/f^ (65, 66) to address how SHP-1 affects Treg function and thereby contributes to T cell homeostasis. While our *ex vivo* studies support a model where SHP-1 negatively regulates the suppressive activity of Treg cells, our *in vivo* models suggest that the role SHP-1 plays in Treg-mediated suppression is more complex. In fact, loss of SHP-1 decreases the effectiveness of Treg cells to suppress inflammation in a model of acute airway inflammation as well as a model of acutely induced autoimmunity. Moreover, mechanistically, our data demonstrate that SHP-1 plays a role in Treg plasticity, AKT-mTOR pathway, and metabolism.

## Materials and methods

### Mice

*SHP1* ^f/f^ mice (65), *Foxp3* ^YFP^-*cre* mice (66), CD45.1 mice (67–69) and *DEREG* mice (70) were purchased from the Jackson Laboratory. CAG-tdTomato Ai14 Foxp3 (71) mice were kindly provided by Dr. Kipnis (Washington University in St. Louis). *SHP1* ^f/f^ mice were crossed to *Foxp3* ^YFP^ *-cre* mice and tdTomato mice to generate *Foxp3* ^YFP^-*cre* x *SHP1* ^f/f^ mice and exTreg lineage-tracing *Foxp3* ^YFP^ *-cre* x *SHP1* ^f/f^ x *tdTomato* mice. Mice used throughout these studies are 6 to 8 weeks old, unless specified otherwise. All experimental mice are age and sex matched and housed and bred in the specific pathogen-free facility at the University of Virginia. Experiments were approved by the Animal Care and Use Committee of the University of Virginia.

### T cell isolation

Lymph nodes and spleens were harvested from naïve 6-8 weeks old mice unless specified otherwise. Tissues were grinded and filtered through 100 µm and 30µm filters. To isolate splenocytes, red blood cell (RBC) lysis was performed using the RBC lysis kit (Invitrogen) according to the manufacturer’s protocol. CD4/CD8 T cells were further isolated using magnetic CD4+ or CD8a+ T cell isolation kits (Miltenyi Biotech) respectively according to the manufacturer’s protocol. Splenocytes were labeled using the kit-provided antibody cocktail and T cells were negatively isolated using the manual LS column. Labeled cells remaining on the column after CD4 isolation were purged out and used as antigen presenting cells following irradiation. Isolated CD4 cells were positively enriched for CD25+ Treg cells using CD25-PE and anti-PE beads (Miltenyi) and for the CD4+CD44+CD25- Tcon isolation, CD4+CD25- cells (derived following CD25 cells depletion) were labeled with CD44-beads (Miltenyi). Populations were isolated *via* a AutoMACS Pro separator (Miltenyi) using Posseld2 program for CD4+ CD25+ Treg and CD4+CD44+ or Deplete program for CD4+CD44- T cells.

### Flow cytometry analysis

Isolated cells were filtered through 30µM separator to get single-cell suspension. Single-cell suspensions were incubated with 2.4G2 Ab (Biolegend) for FcγRII/III blocking and stained with surface staining including: CD3 (BD biosciences/Invitrogen), CD4, CD8 (BD/eBioscience/Biolegend), CD11b, CD137, OX-40, CD28, CD80, CD127, CD45.1, CD45.2 (BD Biosciences), B220, CD279, ICOS, FR4 (Biolegend), CD25 (BD/Biolegend), HELIOS (Invitrogen) in FACS buffer (1% BSA and 0.5mM EDTA). Fixable live/dead staining (Invitrogen) were performed after surface staining in PBS. Cell samples were then fixed in fix/lyse solution (BD Biosciences) or 2% paraformaldehyde for later analysis. For samples stained intracellularly, samples were fixed and permeabilized with BD cytofix/cytoperm kit (BD Biosciences) or Foxp3 transcription factor staining buffer set (eBioscience) according to the manufacturer’s protocol. Intracellular or nuclei staining for pAKT (Cell Signaling), Foxp3 (eBioscience/Invitrogen), ki67 (eBioscience/Biolegend), RORγt, Gata3, Tbet (Biolegend) were performed following the fix/perm treatment. Fixed samples are stored in 4°C until analyzed. Flow cytometry data were acquired on a BD FACSCanto II or Attune Nxt flow cytometer and analyzed using FCS express 7 (research edition) flow software.

### Western blot

Isolated cells with or without stimulation were washed with phosphate-buffered saline (PBS) and lysed with NP-40 buffer (NaCl 150 mM, Tris 50 mM, Nonidet P-40 1%, sodium pyrophosphate 4 mM) or RIPA buffer (NaCl 150 mM, Tris 50 mM, Nonidet P-40 1%, sodium deoxycholate 0.5%, SDS 0.1%) supplemented with a Protease Inhibitors cocktail (Sigma) plus 1mM sodium vanadate, 1mM sodium fluoride, 1 mM PMSF. Lysed samples were spun down at 10,000 g to remove debris and boiled at 100°C for 10 min, with Tris-Glycine buffer (SDS 2%, Bromophenol Blue 0.01%) containing 0.1 M DTT. Aliquots were separated *via* SDS-PAGE. transferred onto PVDF membrane using the Trans-Blot semi-dry transfer system (Bio-Rad). Samples probed for phosphorylation were specially blocked with 5% phosphoBLOCKER (Cell Biolabs) before probing with the following antibodies: anti-SHP-1 (Invitrogen), anti-AKT, or anti-S473-pAKT (Cell Signaling). Blots were re-probed with anti-β-actin HRP (Sigma) to control for loading.

### *In vitro* suppression assay and T cell stimulation

CD4+CD25+ (Treg) cells were isolated from spleens of *Foxp3* ^Cre^ *^+^ Shp-1* ^f/f^ mutant mice or *Foxp3* ^Cre^ *^+^ Shp-1* ^wt/wt^ control mice. Where indicated, we also used *Cre* ^-^ *SHP1* ^f/f^ mice as controls. Since we discovered differences in the levels of Foxp3 protein expression between *Foxp3* ^Cre+^ bearing and Cre^-^ mice (**Supplementary Figure 1C** bottom panel), we included *Foxp3* ^Cre^ *^+^ Shp-1* ^wt/wt^ as controls throughout the studies to normalize for any effect introduced by *Foxp3-Cre*. 2.5x10^4^ CD4+ CD25- Tcon cells from control SHP-1-sufficient mice were labeled with 5µL CellTrace Violet (Life Technologies) and co-cultured with SHP-1-sufficient (control) or SHP-1-deficient (mutant) Treg cells at 1:0, 2:1, 4:1, 8:1, 16:1 and 32:1 ratios in the presence of 150ng/mL anti-CD3 Ab (Cedar Lane Laboratories) in the presence of 5x10^4^ irradiated (2000 rads) CD4+ T cell-depleted splenocytes in 200µL 1640 RPMI complete media (RPMI, Gibco; supplemented with 10% heat inactivated FBS (Seradium), 2mM L-glutamine, 10mM HEPES, 1mM MEM sodium pyruvate, 100µM non-essential amino acids, 50µM 2-mercaptoethanol, and concentration penicillin-streptomycin) in 96-well round bottom plates. After 4 days, cultures were harvested and prepared for flow cytometric analyses. Proliferation was calculated based on CellTrace Violet staining using the proliferation analysis tool in FCS Express research edition. Percentages of suppression were calculated using the following formula:


$$\left[1-\left(\frac{\%divided\;of\;the\;sample}{\%divided\;with\;no\;Treg\;sample}\right)\right]*100\%$$

To stimulate isolated T cells overnight, 96-well plates were coated with goat anti-hamster IgG (5µg/mL, Jackson Lab) and plate-bound anti-CD3 mAb (1µg/mL, Cedarlane Laboratories) overnight before adding 1x10^5^ of the indicated T cells with 2µg/mL soluble anti-CD28 mAb (BD Biosciences) in 200µL RPMI complete media. For experiments that required higher cell numbers, the conditions were scaled up to 24-well plates with 800µL media each.

### Real-time qPCR

Total RNA was extracted from isolated cells using TRIzol reagent and Real-time PCR PureLink-RNA mini kit (Invitrogen). DNA impurities in extracted RNA were removed with DNA-free RNA treatment kit (Applied Biosystems). cDNA was prepared with Applied Biotech cDNA kit according to the manufacturer’s protocols. Quantitative expressions of *shp-1, il-2, il-4, il-10, ebi-3, tgfb-1, tnf, ifng, gapdh*, were measured with TaqMan probes and TaqMan Fast Universal PCR Master Mix (Applied Biosystems) in 96-well plate (US scientific) using QuantStudio 6 Flex system. Relative fold changes were analyzed with QuantStudio Real-Time PCR software, using *gapdh* expression for normalization.

### House dust mite (HDM)-induced allergic airways inflammation (AAI) model

*Foxp3* ^Cre^ *^+^ Shp-1* ^f/f^ mutant mice or *Foxp3* ^Cre^ *^+^Shp-1* ^wt/wt^ control mice from both sexes aged 10 to 12 weeks were used in this 2-week AAI induction model. Mice were anesthetized in isoflurane air flow and then given intranasal instillation. Experimental groups were sensitized intranasally with 10 µg low endotoxin HDM extract (Indoor Biotechnologies) in 50µL sterile PBS while inhaled into the non-AAI control group inhaled PBS at days 0, 2, and 4. During challenge phase, mice were given the same concentration and volume of HDM (or PBS in the non-AAI control group) intranasally at days 10, 12, and14.

At day 16 (24-36 hours after the last challenge), lungs were harvested. To collect BAL fluid, trachea was slit open and flushed with ice cold PBS. BAL fluid was treated with RBC lysis to remove any contaminating red blood cells before flow cytometric analyses. For lung histology, 4% paraformaldehyde fixative was slowly injected through trachea into the lung. To keep lungs inflated, a thread was tied below the opening. Lungs were soaked in the 4% paraformaldehyde for 4 days and processed by the Research Histology Core at the University of Virginia. Samples were embedded in paraffin, sectioned into slides, and stained by H&E or Periodic Acid Schiff (PAS) as indicated. H&E slides were blindly scored by a pathologist following the methods described previously (72). PAS-stained slides were blindly scored using a semi-quantitatively methods as previously described (73), with a modified scale 0-4 representing 0%, 25%, 50%, 75% and 100% of airway epithelium positive for PAS stain.

### Induced autoimmune gastritis (AIG)

DEREG mice (70) were obtained from Jackson Lab. To deplete Treg cells and induce AIG, 12-13 weeks old DEREG mice were treated *via i.p.* injection with Diphtheria Toxin (DT; Calbiochem) at 30 ug/kg body weight in sterile PBS at days 0, 2 and 5 (74). DT concentration had been titrated to limit DT-mediated weight loss to less than 20% during the experimental period. DEREG- littermates were used as control.

Treg cells used for adoptive transfer were isolated from spleens of *Foxp3* ^Cre^ *^+^ Shp-1* ^f/f^ mice or *Foxp3* ^Cre^ *^+^Shp-1* ^wt/wt^ control mice. 0.6 x 10^6^ viable isolated Treg cells in 200 µl PBS were transferred into recipient mice *via* retro orbital injection, an alternative method for tail vein injection introducing less stress to the mice (75), using 30-gauge needles immediately following the first DT injection at day 0. Control mice were injected with 200µl plain PBS solution.

To assess IgE antibody levels, blood was sampled weekly *via* tail vein cut and at the end of the experiment *via* heart puncture. Blood was incubated at 4°C overnight before clearing at 10,000 g. IgE was measured in serum samples (diluted to 1:300) using a mouse IgE ELISA kit (BD biosciences) and 96-well high binding ELISA plates (Corning Costar 9018) according to the manufacturer’s protocol with duplicate data points, and concentrations were calculated based on standard curves.

Mice were euthanized 3 or 5 weeks following the initial DT injection. Stomachs were separated from esophagus and pyloric sphincter, and gastric lymph nodes were collected. Stomachs were sliced open following the inner curvature, and its content was gently washed away with ice-cold PBS. Samples were fixed with Bouin’s fixative solution (RICCA Chemical Company) for 4 days, rinsed with PBS and soaked into 70% ethanol for further slides processing at the Research Histology Core (University of Virginia). Samples were embedded in paraffin, sectioned into slides, and stained by H&E or PAS plus Alcian blue as indicated. Slides were blindly scored with following parameters assessing for levels of gastritis: lymphocytes infiltration, epithelial hyperplasia, and parietal cell loss. All parameters were scored on a scale of 0 - 4 based on severity. For structural changes such as parietal cell loss and mucinous cell hyperplasia, which are possibly associated with loss of gastric function, the score was multiplied by 1.5 adding to a total AIG score between 0 and 16.

### Seahorse analysis of metabolism

Isolated splenic CD4+CD25+ Treg cells from *Foxp3* ^Cre^ *^+^ Shp-1* ^f/f^ mice or *Foxp3* ^Cre^ *^+^ Shp-1* ^wt/wt^ control mice were stimulated (see above) or rested overnight at 37°C in RPMI complete followed by mitochondrial or glycolysis stress tests, which were performed on a Seahorse XF analyzer (Agilent technologies) following the manufacture’s procedure. Briefly, cells were washed and plated in the 96-well seahorse XF cell culture microplates. To measure OCR in mitochondrial stress test, the assay media was composed of non-buffered Seahorse base RPMI media (Agilent technologies), 2mM glutamine (Gibco), 10mM glucose (Sigma) and 1mM sodium pyruvate (Gibco) with a PH adjusted to 7.4. Following measuring baseline OCAR, 1 µM Oligomycin A, 1 µM FCCP and 0.5µM Rotenone & Antimycin A (Sigma) were sequentially added. For glycolysis stress tests, base RPMI media was only supplemented with glutamine. Following measuring baseline ECAR, 10mM Glucose, 1µM Oligomycin and 50 mM 2-DG (Sigma) were sequentially injected. Analyses were performed using WAVE software (Agilent technologies).

### Statistical analysis

Statistical significance was determined using student’s t test (one-tailed/two-tailed and paired/unpaired chosen according to the hypothesis and as indicated in the text) or other pairwise statistic test methods as indicated in text. One-way ANOVA or two-way ANOVA test were performed according to the test requirements. Equal variance was confirmed with residuals vs fit plot. A p-value of<0.05 was considered significant. Asterisks represent *p<0.05, **p<0.01, ***p<0.001, ****p<0.0001.

## Results

### Characterization of *Foxp3* ^Cre+^ *Shp-1* ^f/f^ mice

**Figure 1:**
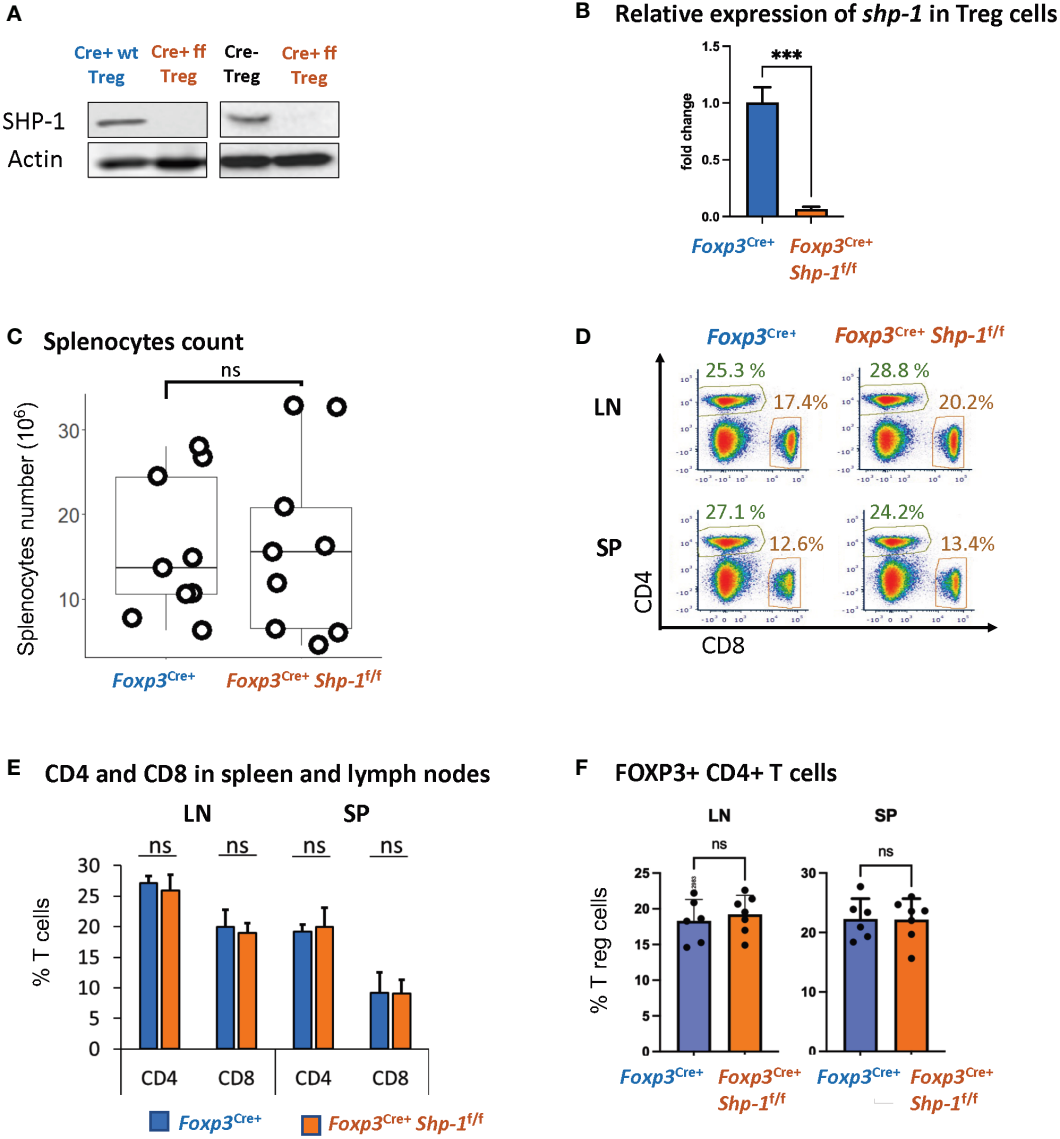
*Foxp3* ^Cre+^ *SHP-1^f/f^ mice display normal T cell composition*. **(A)** SHP-1 protein levels and **(B)** relative *shp1* mRNA expression levels in isolated CD4+ CD25+ Treg as measured by qPCR. *** p< 0.001 **(C)** Total numbers of splenocytes isolated from 6-8 weeks mice of indicated genotypes. Data are from 4 independent experiments with each dot representing one animal. ns, not significant **(D)** Representative flow cytometry data. Percentages of CD4+ and CD8+ subpopulations are indicated. **(E)** Average percentages of CD4+ and CD8+ T cells in lymph nodes and spleens of mice with indicated genotypes. Data are gated: singlets -> live cells. Data points collected from 5 independent experiments with 6 mice of each genotype. **(F)** Percentages of Foxp3+ Treg cells within total CD4+ T cells derived from spleens and lymph nodes of mice with indicated genotypes. n=6 for each genotype. Data are gated: singlets→ live cells → CD3 → CD4. Each dot representing one animal.

We had previously demonstrated that Treg cells derived from mice with a global SHP-1 deficiency, so called *motheaten* mice, exhibited an overall activated phenotype (60). However, as *motheaten* mice have a complex phenotype, we wanted to assess whether SHP-1 has an intrinsic effect on the suppressive function of Treg cells by specifically deleting *shp1* in Treg cells. We crossed mice carrying the floxed alleles of *Ptpn6 (shp1)* (65) with mice that express the Cre recombinase under the control of the *Foxp3* promoter (66). We confirmed highly efficient and specific SHP-1 deletion in CD4+ CD25+ splenic Treg population at protein level (**Figure 1A**) and >99.9% deletion (p value< 0.001) at RNA expression level (**Figure 1B**). CD8^+^ T cells from *Foxp3* ^Cre+^ *Shp-1* ^f/f^ mice showed no detectable SHP-1 deletion (**Supplementary Figure 1A**), suggesting SHP-1 deletion is specific to Foxp3-expressing cells. Importantly, we observed no difference between *Foxp3* ^Cre+^ *Shp-1* ^f/f^ and *Foxp3* ^Cre+^ *Shp-1* ^wt/wt^ control mice in the total splenocytes numbers (**Figure 1C**) and relative CD4+ and CD8+ T cell populations in spleen or lymph nodes (**Figures 1D, E**). Moreover, we aged *Foxp3* ^Cre+^ *Shp-1* ^f/f^ mice under specific pathogen-free condition up to 15-month-old without any overt disease phenotypes emerging.

We had previously observed that due to changes in the thymic selection process, *motheaten* mice have a relative accumulation of Treg cells within the splenic CD4+ T cell compartment (53). However, using this Treg-specific SHP-1 depletion model, there is no difference in the percentage of Foxp3+ Treg cells within the splenic CD4+ population (**Figure 1F**) or levels of Foxp3 expression in Treg cells (**Supplementary Figure 1C** top panel) between *Foxp3* ^Cre+^ *Shp-1* ^f/f^ and control mice. Similarly, there is no difference in the number of Foxp3-expressing thymocytes in mutant and control mice (data not shown). Together, these results suggest that expression SHP-1 protein is not critical for the maintenance of the overall Treg population under steady state conditions.

An earlier report (76) suggested that due to the integration of Cre into the *Foxp3* promoter, there is hypomorphic Foxp3 expression; we also observed a decrease in Foxp3 protein expression in *Foxp3-Cre* ^+^ Treg cells, independent of SHP-1 expression (**Supplementary Figure 1C** bottom panel). In addition, we observed increased total splenocytes numbers (**Supplementary Figure 1B**) and relative percentages of Foxp3^+^ Treg cells within the CD4+ T cell population of *Foxp3* ^Cre+^ *Shp-1* ^f/f^ mice (**Supplementary Figure 1E**). However, relative CD4+ and CD8+ subpopulations remained comparable (**Supplementary Figure 1D**). Decreased Foxp3 level have previously been linked to lower suppression function (77) and increased Treg proliferation (78, 79) *in vivo.* To avoid any confounding issues from *Foxp3-Cre*, we have used *Foxp3* ^Cre+^ *Shp-1* ^wt/wt^ as control in all the studies described below.

### Characterization of Treg population within *Foxp3* ^Cre+^ *Shp-1* ^f/f^ mice

**Figure 2:**
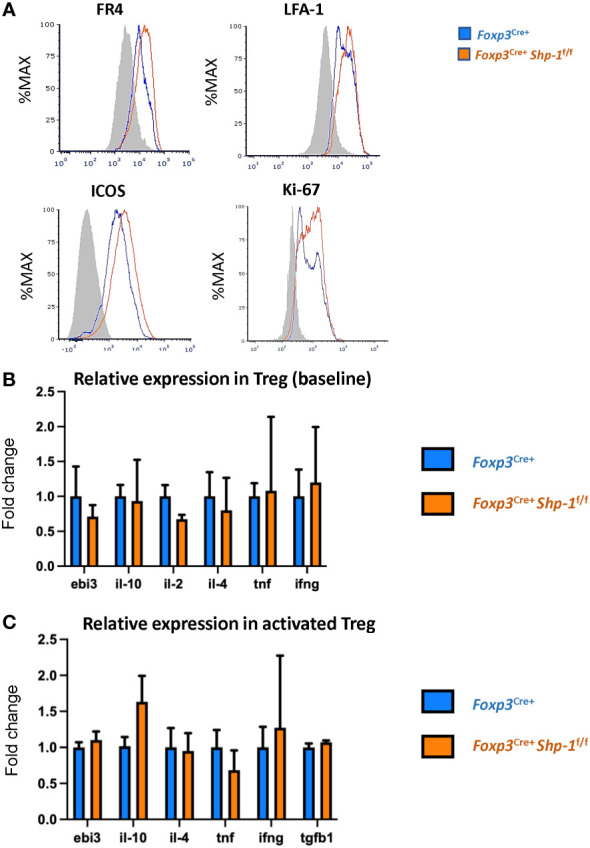
*Phenotypic analyses of 6-8 weeks old Foxp3^Cre+^ Shp-1^f/f^ and Foxp3^Cre+^ control mice*. **(A)** Histograms depict representative protein expression levels of live splenic CD4+ FOXP3+ cells as assessed by flow cytometric analyses. Data are gated: singlets→ live cells → CD3 → CD4 → Foxp3. Statistical analyses of the MFIs (geometric mean) were performed using a two-way ANOVA test (FR4 – p=0.03878; LFA-1 – p=0.0154; ICOS – p=0.0133; Ki67 – p=0.0096) (**B**, **C**) Relative cytokine expression of **(B)** freshly isolated and **(C)** stimulated splenic CD4+CD25+ Treg cells isolated from 6-8 weeks old mice of the indicated genotypes, **(B)** n = 3 for each genotype. **(C)** n= 5 for stimulated Treg. **(C)** Treg cells were incubated for 14-18 hrs. with plate bound anti-CD3 (150 ng/ml) and soluble anti-CD28 (500 ng/ml). Error bars represent SD.

Next, we asked how the Treg population was affected by the loss of SHP-1 protein. When we analyzed proteins linked to Treg cell function and/or activation status (80–82), we observed slightly higher, yet statistically significant levels of folate receptor 4 (FR4), lymphocyte function-associated antigen 1 (LFA-1) and inducible T-cell costimulator (ICOS) protein expression on SHP-1-deficient Treg cells (**Figure 2A**) in the *Foxp3* ^Cre+^ *Shp-1* ^f/f^ mice. Moreover, *Foxp3* ^Cre+^ *Shp-1* ^f/f^ Treg cells appeared more proliferative *in vivo* as measured by Ki-67 staining (**Figure 2A**). However, several Treg markers remained unchanged (CD54, CD80, OX-40, LAG-3, CD103, CD137, CD28, PD-1, CD127) or with a trend of slight increase (such as CD25, CTLA-4, GITR) in *Foxp3* ^Cre+^ *Shp-1* ^f/f^ mutant mice compared to *Foxp3* ^Cre+^ *Shp-1* ^wt/wt^ control mice at steady state (**Supplementary Figure 2A**). Moreover, there was no spontaneous induction of a skewed T-bet, Gata-3 or RORγt-expressing Treg subpopulation in the *Foxp3* ^Cre+^ *Shp-1* ^f/f^ mutant mice (**Supplementary Figure 2B**). Finally, there were no significant differences in cytokine expression in *Foxp3* ^Cre+^ *Shp-1* ^f/f^ Treg cells basally, with or without CD3/CD28 stimulation besides a trend of IL-10 increase in stimulated *Foxp3* ^Cre+^ *Shp-1* ^f/f^ Treg cells, (**Figure 2B** and **Supplementary Figure 2C**). Thus, freshly isolated SHP-1-deficient Treg cells display certain features of an activated phenotype but show normal expression of numerous other Treg markers compared to control Treg cells.

### Treg-specific SHP-1 deletion leads to greater AKT activation

**Figure 3:**
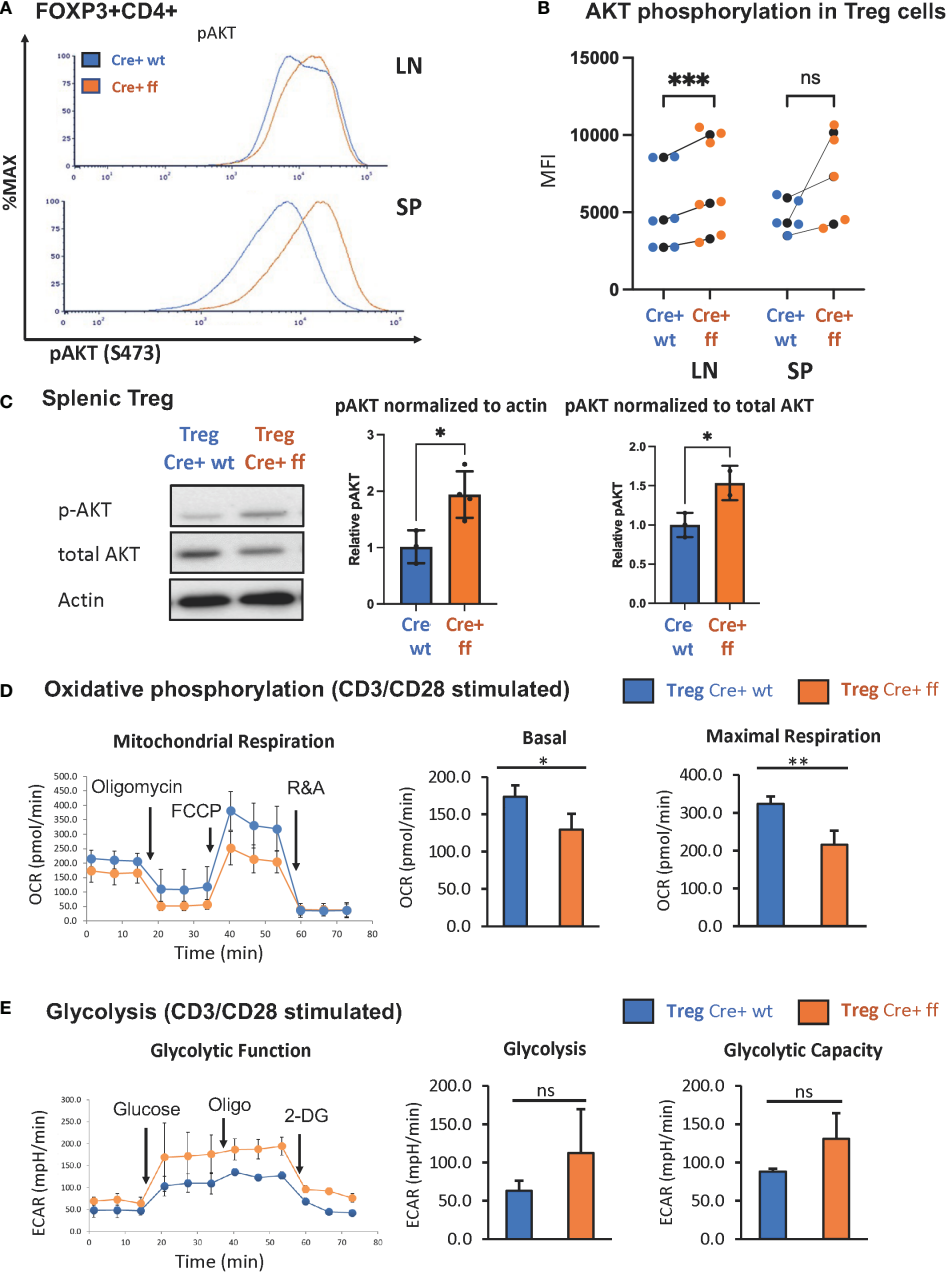
*Treg specific SHP-1 deletion increases phosphorylation of AKT and affects cellular metabolism*. **(A)** AKT phosphorylation (Ser473) in freshly isolated Treg of 6-8 weeks old mice. Data are representative of 3 independent experiments. **(B)** MFIs of pAKT of *Foxp3* ^Cre+^ *Shp-1* ^f/f^ Treg cells and *Foxp3* ^Cre+^ control Treg cells from lymph node or spleen were plotted. Each colored dot represents one data point. Average values for each genotype from the same experiment are shown as black dot and matched by lines for each experiment. Two-way ANOVA test was performed for statistics. LN p-value = 0.0005, SP p-value = 0.0521. Data are gated singlets → live cells → CD3 → CD4 → Foxp3 **(C)** Immunoblot analysis of p-AKT(S473), total AKT and β-actin from control *Foxp3* ^Cre+^ (“cre wt”) and *Foxp3* ^Cre+^ *Shp-1* ^f/f^ (“cre ff”) mutant Treg cells stimulated with anti-CD3/CD28 beads + IL-2 (200U/ml) for 5 min. Relative pAKT quantity were normalized to actin and total AKT. Unpaired t test was used for statistics. pAKT/actin p-value = 0.0220, pAKT/total AKT p-value =0.0464. **(D, E)** CD4+CD25+ Treg cells of *Foxp3* ^Cre+^ *Shp-1* ^f/f^ or *Foxp3* ^Cre+^ control mice were stimulated with CD3/CD28 Dyna beads according to the manufacture’s protocol overnight and using a Seahorse bioanalyzer. **(D)** Basal and maximal mitochondrial respiration and **(E)** glycolysis and glycolysis capacity were measured to assess **(D)** OCR and **(E)** ECAR respectively, n=3 for each genotype. Error bar represents **(B)** SD and (**D**, **E**) s.e.m., two-sided t test, *p<0.05, **p<0.01, ***p<0.001, ns, not significant.

Previous studies from our lab demonstrated that the SHP-1 inhibits the activation of the PI3K/AKT pathway in CD4+ Tcon cells (62). Moreover, PI3K/AKT activation has been linked to promoting proliferation in Tregs (83). As we noted that SHP-1 deficiency increased the percentage of Ki-67+ Treg cells indicating augmented proliferation *in vivo*, we explored whether the *Foxp3* ^Cre+^ *Shp-1* ^f/f^ Treg cells display an enhanced PI3K/AKT pathway activation. AKT phosphorylation at S473 was used as a surrogate measurement of AKT activation. Freshly isolated splenic *Foxp3* ^Cre+^ *Shp-1* ^f/f^ Tregs displayed an increased AKT phosphorylation compared to *Foxp3* ^Cre+^ and *Shp-1* ^f/f^ control Treg cells. Although not statistically significant when compared to *Foxp3* ^Cre+^, a trend towards an increase was also observed in lymph node derived Treg cells (**Figures 3A, B** and **Supplementary Figures 3A, B**). This increase was even more pronounced upon *in vitro* anti-CD3/anti-CD28 stimulation (**Figure 3C** *).* Interestingly, we consistently observed a trend toward a small but not statistically significant decrease in total AKT protein levels in the mutant Treg cells despite the hyperphosphorylation. Together these data suggest that SHP-1 limits signaling along the PI3K/AKT/mTOR pathway in Treg cells.

We next asked whether the increased AKT phosphorylation may translate to a metabolic change, as AKT activation is linked to increased glycolytic activity (78). While the freshly isolated unstimulated Treg cells showed no significant oxygen consumption rate (OCR) difference between the *Foxp3* ^Cre+^ *Shp-1* ^f/f^ mutant and *Foxp3* ^Cre+^ *Shp-1* ^wt/wt^ control Treg cells (**Supplementary Figure 3C**), TCR/CD3 stimulation showed decreased mitochondrial respiration, as measured by the OCR at both basal and maximal level (**Figure 3D**) in SHP-1-deficient Treg cells. Moreover, *Foxp3* ^Cre+^ *Shp-1* ^f/f^ Treg cells displayed a trend toward increased extracellular acidification rate (ECAR) compared to control Treg cells, suggesting higher glycolytic activity (**Figure 3E**). It should be noted that the link between AKT phosphorylation status and Treg cell plasticity and function is complex (84), and we address the Treg cell lineage stability and functionality in the *Foxp3* ^Cre+^ *Shp-1* ^f/f^ mice further below.

### Treg-specific SHP-1 deletion affects other T cell populations *in vivo*

**Figure 4:**
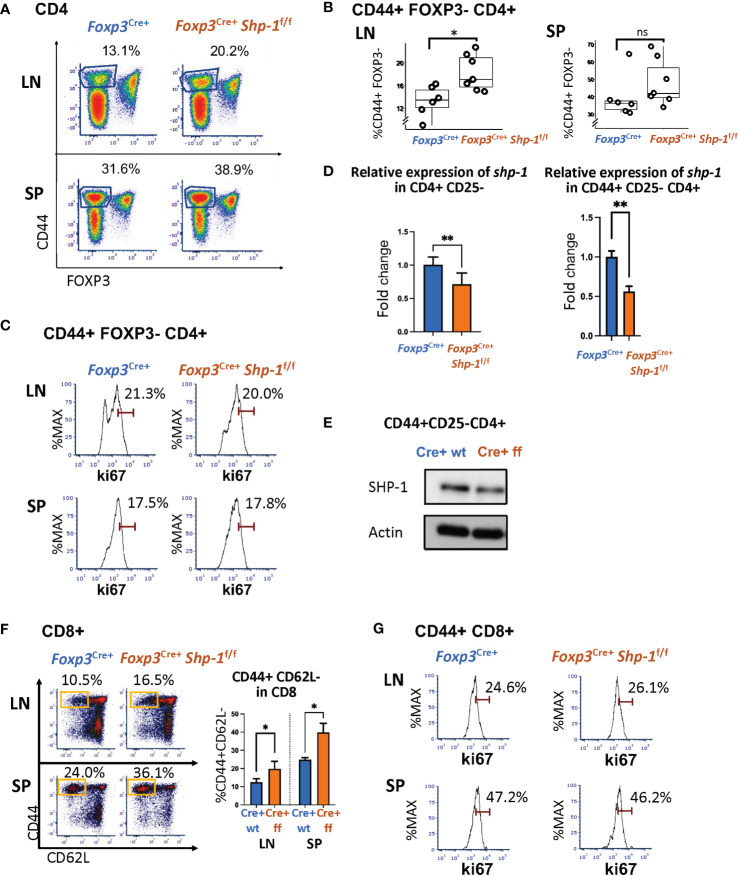
*Treg specific SHP-1 deletion affects non-Treg T cell population.* **(A**, **B)** Percentages of CD44^hi^ Foxp3- within CD4+ T cell population of lymph nodes and spleen of *Foxp3* ^Cre+^ *Shp-1* ^f/f^ or *Foxp3* ^Cre+^ mice. **(A)** Dot Plot is representative of 5 independent experiments. Percentages of CD44+ Foxp3- within CD4 populations are indicated. **(B)** Each dot represents one animal; lymph node p-value = 0.01, spleen p-value = 0.2537, two-sided t test. Gated: singlets → live → CD4. Student T test were performed for statistics. **(C)** Percentages of Ki-67+ within CD44^hi^ Foxp3- CD4+ T cells of lymph nodes and spleen of *Foxp3* ^Cre+^ *Shp-1* ^f/f^ or *Foxp3* ^Cre+^ mice. Data are representative of 3 independent experiments. Gated: Gated: singlets → live → CD4 → FOXP3- → CD44. (**D** *left panel*) CD4+ CD25- cells or (***D*** *right panel* and **E**) CD4+ CD44^hi^ CD25- cells were magnetically sorted from *Foxp3* ^Cre+^ *Shp-1* ^f/f^ or *Foxp3* ^Cre+^ control mice and assessed for **(D)** mRNA expression via quantitative RT-PCR analysis of *shp-1* mRNA (n =3 for each genotype, p-value for left panel = 0.0017, p-value for right panel = 0.0067) and for **(E)** SHP-1 protein expression via immunoblotting. Percentages of **(F)** CD44^hi^ CD62L^lo^ (n= 3-4 for each genotype, p-value for LN=0.0379, p-value for SP=0.0282) and **(G)** Ki-67+ T cells within CD44+ CD8+ T cell population of lymph nodes and spleen of *Foxp3* ^Cre+^ *Shp-1^f^* ^/f^ or *Foxp3* ^Cre+^ mice. Data are representative of 3 independent experiments. Gated: **(C)** singlets → live → CD4 → FOXP3- →CD44. **(F)** singlets → live → CD8. **(G)** singlets → live → CD8 →CD44. Unpaired t test. *, p<0.05, **, p<0.01. (**D**, **E**) CD4+ CD44^hi^ CD25- cells were magnetically sorted from *Foxp3* ^Cre+^ *Shp-1* ^f/f^ or *Foxp3* ^Cre+^ control mice and assessed for SHP-1 protein levels and mRNA expression *via* **(D)** Immunoblot and **(E)** Quantitative RT-PCR analysis of *shp-1* mRNA. Percentages of **(F)** CD44^hi^ CD62L^lo^ and **(G)** Ki-67+ T cells within CD8+ T cell population of lymph nodes and spleen of *Foxp3* ^Cre+^ *Shp-1^f^* ^/f^ or *Foxp3* ^Cre+^ mice. Data are representative of 3 independent experiments. Gated: singlets → live → CD8. ns, not significant.

Next, we asked whether the Treg-specific loss of SHP-1 resulted in phenotypic changes in the non-Treg lymphocyte populations. We observed a significant increase in the CD44^hi^CD62L^lo^ antigen-experienced CD4+ T cells within the non-Treg CD4+ Tcon cell compartment derived from lymph nodes of naïve *Foxp3* ^Cre+^ *Shp-1* ^f/f^ mutant mice as well as a trend towards an increased CD44^hi^CD4+ splenic T cell population in mutant mice (**Figures 4A, B** and **Supplementary Figure 4**). This CD4+ CD44^hi^ Tcon cell population from *Foxp3* ^Cre+^ *Shp-1* ^f/f^ mutant express Ki-67 at levels comparable to control mice, indicating similar degrees of proliferation (**Figure 4C**). However, an analysis of the *shp-1* mRNA expression levels in CD25-CD4+ Tcon and the CD44^hi^ CD25-CD4+ T cell subsets derived from *Foxp3* ^Cre+^ *Shp-1* ^f/f^ mutant mice showed a significant decrease compared to control mice (**Figure 4D**), which was also confirmed at the protein level (**Figure 4E**), suggesting that at least some of the CD44^hi^ antigen experienced populations in the CD4+ Tcon cell compartment might be exTreg cells, which we address below.

Interestingly, CD8+ T cells isolated from spleen or lymph nodes of naive *Foxp3* ^Cre+^ *Shp-1* ^f/f^ mice also contain higher CD44^hi^CD62^lo^ subpopulations (**Figure 4F**), indicating a basal increase in antigen-experienced CD8+ T cells. As we had observed for the CD4+ T cell lineage, Ki-67 staining of the CD44^hi^ CD8+ T cell population was comparable between *Foxp3* ^Cre+^ *Shp-1* ^f/f^ mutant and control mice (**Figure 4G**), indicating that the overall increase in CD44^hi^ non-Treg T cells is not driven by hyper-proliferation. We confirmed the lineage specificity of *Foxp3* ^Cre+^ by assessing *shp1* mRNA levels in the CD8+ T cell population using RT-PCR, a population that should not be directly affected by *Foxp3* ^Cre+^ and found comparable *shp1* mRNA levels in CD8 cells derived from mutant and control mice (**Supplementary Figure 1A**) suggesting that SHP-1-deficient Treg cells affect the phenotype of the CD8 T cell lineage.

### Treg-specific SHP-1 deletion increases ex-Treg compartment *in vivo*

**Figure 5:**
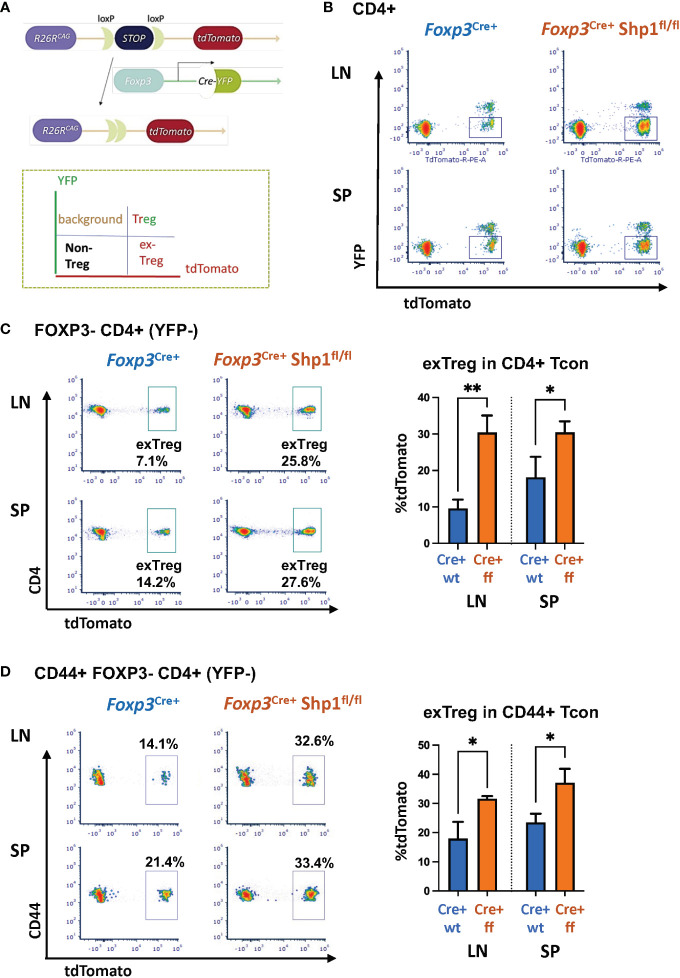
*SHP-1 promotes Treg cell lineage stability*. **(A)** Schematic diagram and **(B)** flow cytometry representation of Treg lineage tracing model. Gates for tdTomato+ YFP- (exTreg) cells are indicated throughout the panels. tdTomato (cre-inducible expression) were crossed with *Foxp3* ^YFP-cre^/*Shp-1* ^f/f^ and *Foxp3* ^YFP-cre^ control mice. **(C**, **D)** Plot depicts T cell subpopulations based on YFP and tdTomato expression. Flow cytometric analyses of **(C)** tdTomato+ (exTreg) within CD4+ FOXP3 (YFP)- Tcon (LN p-value =0.0023, SP p-value = 0.0449) and **(D)** tdTomato+ (exTreg) within CD44+FOXP3-CD4+ T cells in 8 weeks old mice (LN p-value = 0.0146, SP p-value = 0.0388). Percentages of exTreg cells within each subpopulation are indicated. Gated: **(B)** singlets → live → CD4 **(C)** singlets → live → CD4 → FOXP3-(YFP-) **(D)** singlets → live → CD4 → FOXP3-(YFP-) →CD44. Data are from 2 experiments with each 2-3 mice per genotype. *, p<0.05, **, p<0.01.

As some of the CD4+ CD44^hi^ Tcon cells seen above might represent the previously reported ‘ex-Treg cells’ that have lost Foxp3 expression, we next asked whether SHP-1 may help maintain Treg cell lineage stability. To test this, we turned to a lineage tracing model that expresses a Cre-inducible tdTomato by crossing *Foxp3* ^YFP-Cre+^ *Shp-1* ^f/f^ or *Foxp3* ^YFP-Cre+^ *Shp-1* ^wt/wt^ onto the *Rosa26* ^tdTomato^ strain (68) (**Figure 5A**). We expected that if the CD44^hi^ Tcon cells were to be ex-Treg cells, then the Foxp3-Cre would have been active in these cells at one point, leading to tdTomato expression, even if they have since lost the Cre-YFP expression. This can then be distinguished from the non-Treg-derived Tcon cells (which would be negative for both tdTomato and YFP fluorescence) and the Treg cells (which continue to express both tdTomato and YFP) (**Figure 5B**). We found that loss of SHP-1 in Treg cells caused an increase in exTregs (tdTomato^+^ but YFP^-^) within the CD4+ Tcon cell population in both the spleen and lymph nodes (**Figure 5C**). This suggested that SHP-1-deficient Treg cells might be less stable leading to an accumulation of an exTreg population. It is noteworthy that not all CD4+ CD44^hi^ Tcon cells are exTreg cells, but that the population is enriched in the *Foxp3* ^YFP-Cre+^ *Shp-1* ^f/f^ mice compared to *Foxp3* ^YFP-Cre+^ control mice (**Figure 5D**). This suggests that the overall increase in the CD44^hi^ antigen-experienced population in *Foxp3* ^YFP-Cre+^ *Shp-1* ^f/f^ mice might come from both reduced Treg stability as well as additional secondary factors leading to further accumulation of the CD4+ CD44^hi^ Tcon population.

### SHP-1-deficient Treg cells demonstrate increased suppressive activity *in vitro*

**Figure 6:**
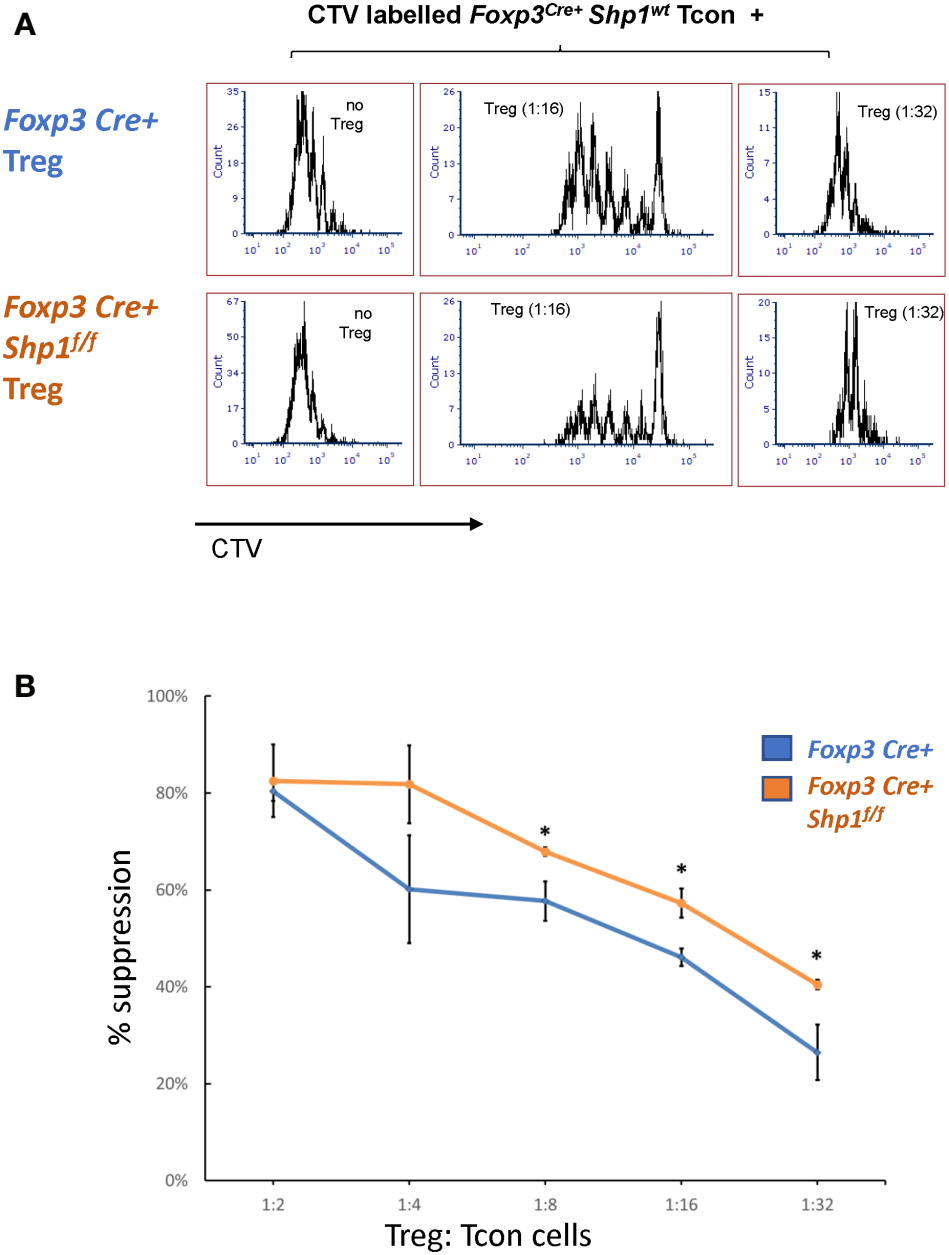
*SHP-1-deficient Treg cells display increased suppressive activity in vitro*. **(A**, **B)** Treg (CD4+CD25+ isolated from *Foxp3* ^Cre+^ *Shp-1^f^* ^/f^ mutant or *Foxp3* ^Cre+^ control mice) and CTV-labeled Tcon (CD4+CD25- from control mice) cells were co-cultured at the indicated ratios. **(A)** Histogram depicts CTV-dilution of Tcon cells as a measurement of proliferation. Data are representative of 3 independent experiments with each n= 1 - 3 mice (6-9 weeks) per genotype. **(B)** Suppression capacity of Treg cells based on data obtained in **(A)** Error bar represents SD. *, p≤ 0.05.

To address whether SHP-1-deficient Treg cells are functional, we first tested the suppressive capacity *ex vivo*. Using *in vitro* suppression assays, SHP-1-deficient Treg cells showed increased suppressive capacity toward wild type CD4+ Tcon cells compared to *Foxp3* ^Cre+^ or *Shp-1* ^f/f^ control Treg cells, which was most evident at lower Treg : Tcon ratios (**Figures 6A, B** and **Supplementary Figures 5A, B**).

To assess whether SHP-1-deficient Treg cells might hyper-proliferate in response to IL-2, we stimulated Treg cells in culture with 0, 20, 200 and 2000 Unit of IL-2 for 4 days. Both *Foxp3* ^Cre+^ control and *Foxp3* ^Cre+^ *Shp-1* ^f/f^ mutant Treg proliferated at similar levels when given medium to high level of IL-2 stimulation, while mutant Treg cells show less proliferation at low or no IL-2 condition (**Supplementary Figure 5C** left). Treg from both genotypes show comparable viability and maintain similar percentages of Foxp3+ cells within the culture under the same IL-2 concentration (**Supplementary Figure 5C** right), demonstrating that the observed increase in suppressive activity mediated by SHP-1-deficient Treg cells is not due to increased Treg proliferation in an *in vitro* setting. These data indicate that SHP-1-deficient Treg cells have the potential not only to be functional but are hyper-suppressive in a short-term *ex vivo* setting. However, we interpret these *ex vivo* results with caution, as it is well recognized that Treg functionality measured *in vitro* may not reflect the larger Treg functionality/biology *in vivo*.

### Mice with SHP-1-deficient Treg cells show impaired control of inflammation *in vivo*

**Figure 7:**
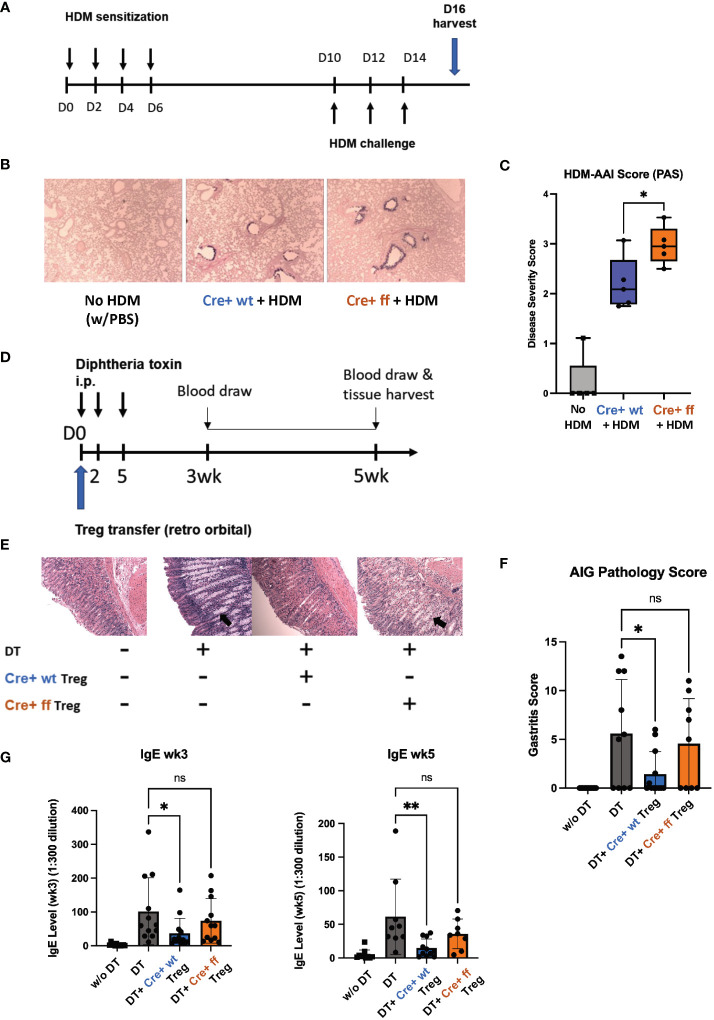
*SHP-1 is required for the suppressive functionality of Treg cells in vivo.* **(A)** Schematic diagram depicts HDM-AAI experimental setup. **(B)** Representative images of lung histology (PAS/Alcian blue staining) of *Foxp3* ^Cre+^ control and *Foxp3* ^Cre+^ *Shp-1* ^f/f^ mice 5 weeks after HDM or PBS treatment. Magnification: 4x **(C)** HDM-induced AAI disease severity represented by score based on PAS stained mucous. n= 6 (*Foxp3* ^Cre+^ control), 6 (*Foxp3* ^Cre+^ *Shp-1* ^f/f^ mutant), 5 (PBS treated no HDM), **(D)** Schematic diagram of DEREG model. Treg cells are depleted by consecutive Diphtheria toxin (DT) injections causing an AIG phenotype that can be rescued *via* adoptive transfer of Treg cells. **(E)** Representative images of stomach histology (H&E staining) of indicated experimental groups. Magnification: 10X. Arrows indicate epithelia hyperplasia/metaplasia. **(F)** AIG disease severity score based on H&E and PAS-stained histology images. n= 12 (no DT), 10 (DT), 12 (*Foxp3* ^Cre+^ control), 9 (*Foxp3* ^Cre+^ *Shp-1^f^* ^/f^ mutant). ns = not significant. **(G)** Serum IgE level of the indicated experimental groups at 3 and 5 wks. Data are from three independent experiments with 2-3 mice per experimental group for each experiment. *, p<0.05, **, p<0.01. One-way ANOVA, followed by Fisher’s LSD test **(C**, **G)**, or Brown-Forsythe and Welch ANOVA test followed by Welch test **(F)** for multiple comparisons. ns, not significant.

We next tested the functionality of SHP-1-deficient Treg cells *in vivo* by comparing the abilities of SHP-1-deficient and -sufficient Treg cells to control inflammation *in vivo*. First, we employed a model of allergic airways inflammation (AAI), which is acutely induced through exposure to the common allergens from the house dust mite (HDM) (**Figure 7A**) (72). A comparison of mutant and control mice following HDM treatment showed higher lung mucous production in *Foxp3* ^Cre+^ *Shp-1* ^f/f^ mutant mice (**Figure 7B**), when assessed by a semiquantitative severity score (73) based on PAS stain (**Figure 7C**). This suggested that Treg cells in *Foxp3* ^Cre+^ *Shp-1* ^f/f^ mice have a decreased capacity to suppress inflammation in the AAI model *in vivo*, despite their greater ability to suppress *in vitro*. Nevertheless, there were no significant differences in the T cell populations derived from draining lymph nodes, bronchoalveolar lavage fluid, or lung tissues and consistent with this finding, the levels of bronchiolar and vascular inflammation were comparable (**Supplementary Figure 6A**). This decrease in suppressive ability was surprising, as we had initially expected the greater suppressive activity *in vitro* to translate to reduced disease severity *in vivo*.

To complement the AAI model, where inflammation is induced in response to a foreign antigen, we employed the DEREG (DEpletion of REGulatory T cells) model, in which mice develop inflammatory disease in the form of autoimmune gastritis (AIG) upon Treg cell depletion (74). The phenotype can be rescued through adoptive transfer of Treg cells allowing to compare the suppressive capacities of Treg cells in a genetically identical background (**Figure 7D**) (70). Diphtheria toxin (DT)-mediated Treg depletion has been shown to cause a Th2-skewed immune response as evidenced by elevated serum IgE level (74). AIG-induced mice rescued with *Foxp3* ^Cre+^ *Shp-1* ^wt/wt^ control Treg cells showed a reduced inflammation as reflected by pathology score, with reduced lymphocyte infiltration, epithelia hyperplasia/metaplasia, and parietal cell loss (**Figure 7E**). However, transfer with *Foxp3* ^Cre+^ *Shp-1* ^f/f^ Treg cells failed to rescue the AIG pathology score (**Figures 7E, F**). With respect to serum IgE levels, at 3- and 5-week post transfer, adoptive transfer of wild type Treg cells significantly reduced the serum IgE levels, while *Foxp3* ^Cre+^ *Shp-1* ^f/f^ Treg cells failed to do so (**Figure 7G**). In the DEREG induced AIG model, host-derived Treg cells reappear within a few days following DT-mediated Treg depletion (74) *(* **Supplementary Figure 6B** *left panel)*. To precisely distinguish between host and donor Treg cells, we crossed the B6 CD45.1 allele mice (67–69) onto the DEREG background. We found comparable levels of donor *(CD45.1- CD45.2+) Foxp3* ^Cre+^ *Shp-1* ^f/f^ mutant and *Foxp3* ^Cre+^ control Treg cells *(* **Supplementary Figure 6B**, *middle panel*), and both experimental groups limited the reappearance of host-derived Treg cells (CD45.1+CD45.2+) at a comparable level (**Supplementary Figure 6B** *, right panel)* confirming that there was no difference between the adoptive transfer of the two experimental groups. It also indicates that this early effect homeostatic expansion of the host Treg population was not affected by the loss of SHP-1 in the donor Treg cells.

Since we had observed that loss of SHP-1 causes an instability of the Treg lineage commitment (**Figure 5B**), we next assessed whether adoptively transferred donor Treg cells are localized to the site of inflammation. 3 weeks after the adoptive Treg transfer, there were no detectable donor *Foxp3* ^Cre+^ *Shp-1* ^f/f^ cells (CD45.2+ CD45.1-) in the host gastric draining lymph nodes, while in the *Foxp3* ^Cre+^ control donor group, about 1% of the CD45+ leukocytes were donor-derived. Within this population, about 70-80% remained Foxp3+ (**Supplementary Figures 6C, D**). These finding suggest that although *Foxp3* ^Cre+^ *Shp-1* ^f/f^ Treg cells are initially able to control the homeostatic expansion of host Treg cells, they fail to sufficiently migrate and/or survive at the site of inflammation in the *in vivo* AIG model. Neither *Foxp3* ^Cre+^ *Shp-1* ^f/f^ nor *Foxp3* ^Cre+^ control donor group-derived cells could be detected in the spleen 3 weeks post transfer. This failure of SHP-1-deficient Treg cells to efficiently control inflammation in two different models of inflammatory disease suggests a critical role for SHP-1 in the *in vivo* functionality of Treg cells.

## Discussion

Our findings, based on Treg-specific deletion of SHP-1, suggest previously unappreciated roles for this tyrosine phosphatase in regulating Treg function and homeostasis. While the loss of SHP-1 *via* the *Foxp3* ^Cre+^ *Shp-1* ^f/f^ approach does not quantitatively affect the thymic Treg selection process under these conditions, there are qualitative differences between *Foxp3* ^Cre+^ control and *Foxp3* ^Cre+^ mutant Treg cell populations. Interestingly, despite the increased suppressive activity of SHP-1-deficient Treg cells *in vitro*, the Treg cells appear less stable *in vivo* and tend to lose Foxp3 expression, and a greater fraction of them transition to ex-Treg cells at steady state. Further, when *Foxp3* ^Cre+^ *Shp-1* ^f/f^ mice were challenged in inflammatory models (either exogenous antigen or autoimmune settings), the Treg cells fail to effectively control the inflammation. Thus, SHP-1 has critical functions in Treg cells during the mitigation of inflammation.

Our data demonstrate an increase in the suppressive activity of SHP-1-deficient Treg cells *in vitro*. However, when assessed *in vitro*, the expression of suppressive cytokines such as IL-10 or IL-35 (**Figures 2B, C**) were comparable between mutant and control Treg cells suggesting SHP-1 regulates Treg cell suppressive activity through mechanisms other than suppressive cytokine productions. Further, while a higher percentage of *Foxp3* ^Cre+^ *Shp-1* ^f/f^ Treg cells are proliferating *in vivo* based on Ki-67 expression levels (**Figure 2A**), in the *in vitro* suppression assay, Treg numbers from both *Foxp3* ^Cre+^ *Shp-1* ^f/f^ and control groups are comparable after 4 days in culture (data not shown). *In vitro* suppression assays measure the potential suppressive capacity of Treg cells under optimal conditions. As these assays only partially reflect the *in vivo* setting, which integrates additional factors that directly and indirectly modulate Treg-mediated suppression, including trafficking and maintenance of Treg cells, we utilized two mouse models: the HDM antigen-induced AAI model and DT-induced AIG model. In both models, *Foxp3* ^Cre+^ *Shp-1* ^f/f^ Treg performed worse when challenged and failed to protect the host from both allergic response and autoimmune disease.

In the AAI model, the discrepancy between *in vitro* and *in vivo* suppressive function might be partially explained by the resistance to Treg suppression arising from the Tcon compartment: *Foxp3* ^Cre+^ *Shp-1* ^f/f^ mice contain more CD44^hi^ CD62L^low^ CD4+ T cell population (**Figure 4A** and **Supplementary Figure 4**), which expresses lower levels of SHP-1 (**Figure 4D**) and potentially rise from a subpopulation of SHP-1-deficient ex-Treg cells. We have previously shown that loss of SHP-1 in Tcon cells promotes resistance to suppression (62). At steady state, *Foxp3* ^Cre+^ *Shp-1* ^f/f^ mice have thymic (data not shown) and peripheral cell numbers as well as CD4+, CD8+, Foxp3+ T cell frequencies (**Figure 1E**) comparable to control mice and exhibit no spontaneous disease phenotypes. This data suggests both central and peripheral tolerance are maintained at homeostatic conditions. However, upon challenge, this otherwise dormant CD44^hi^ CD62L^low^ population may not only be less suppressible, but also more readily activated upon any inflammatory signals to carry out a robust response.

In the transient Treg-depletion in the DEREG system, the host Treg cells quickly come back, although they are not able to control autoimmune gastritis. Interestingly, these quickly resurrected host Treg cells exhibit normal *in vitro* suppressive capacity (74). The reappearance of the host Treg cells is reduced upon adoptive transfer of Treg cells. In our studies, we detected comparable levels of returning host Treg cells in spleen and lymph nodes upon transfer of SHP-1-deficient or -sufficient Treg cells indicating that this Treg-mediated control of homeostasis is unaffected by the presence of absence of SHP-1 (**Supplementary Figure 6B**). However, the transferred SHP-1-deficient Treg cells failed to control AIG, while control Treg cells were able to limit AIG. Although *Foxp3* ^Cre+^ *Shp-1* ^f/f^ Treg can be found at similar level as the Treg from control mice in host blood on day 4 after the transfer. At 3 weeks post transfer, the SHP-1-deficient Treg cells are not detectable in the gastric draining lymph nodes (**Supplementary Figures 6C, D**), while *Foxp3* ^Cre+^ control Treg cells are maintained as a small, but functionally significant population. The inability of *Foxp3* ^Cre+^ *Shp-1* ^f/f^ Treg cells to suppress AIG, is not due to the loss of Foxp3 expression or conversion to ex-Treg cells, since no *Foxp3* ^Cre+^ *Shp-1* ^f/f^ donor cells were detectable at 3 weeks, regardless of Foxp3 expression. Impaired Treg functions during recruitment, retention or survival at stomach mucosa may contribute to disappearance of transferred SHP-1-deficient Treg cells. Interestingly, it has previously been reported that SHP-1 deficient Treg cells might actively contribute to an increased response to food-mediated allergies via a reprogramming towards a TH2 phenotype (85). While in all of these studies, SHP-1-deficient Treg cells were inefficient in preventing/mitigating inflammatory diseases, the underlying mechanisms are likely different.

Similar to our previous findings in Tcon cells (62), SHP-1 regulates AKT phosphorylation in Treg cells. The role of AKT activation and the AKT-mTOR pathway in Treg cells is complex. Early studies noted strong TCR activation promotes AKT phosphorylation, which then inhibits FOXO transcription factors promoting FOXO localization out of the nucleus thereby inhibiting the generation of peripherally induced Treg cells (78, 86–89). These studies either stimulated the AKT/mTOR pathway through TLR (78), rapamycin (86), used TCR agonist peptides in TCR transgenic T cells (87, 90), or Raptor knockout mice with strong autoimmune phenotype (87). Although these results seem to contrast our findings that *Foxp3* ^Cre+^ *Shp-1* ^f/f^ Treg display a higher AKT phosphorylation while also exhibiting a stronger suppression *in vitro*, it is worth noting that conditional SHP-1 deletion as done in our study presents a more modest modulation of TCR activation, which does not affect the overall Foxp3 expression and may not impair the Treg suppressive function.

*Foxp3* ^Cre+^ *Shp-1* ^f/f^ mice show an expanded CD44^hi^ CD62L^lo^ CD4+ Tcon population (**Figure 4A** and **Supplementary Figure 4**). A similar increase of antigen-experienced cells in the CD4 and CD8 compartments had previously been reported by Johnson et al. in CD4-Cre SHP1^f/f^ mice (91). We have previously performed extensive series of studies in our lab using dLck-Cre SHP-1^f/f^ mice but did not find a similar effect (62). A possible explanation for these differences might be the timing of the SHP-1 depletion. While CD4-Cre and Foxp3-Cre already drive Cre expression at the double positive stage during the thymic selection process, Cre driven by the distal LCK promoter is expressed post-selection starting at the single positive stage (92) allowing for physiological SHP-1 expression during thymic selection. The TCR repertoire generated during thymic T cell development is the product of positive and negative selections and is directed by the strength of signaling downstream of the TCR, a signaling pathway regulated by SHP-1. SHP-1 has been shown to be crucial for the differentiation between low-affinity versus high-affinity altered peptide ligands using OT-I *CD4-Cre SHP1* ^f/f^ transgenic mice (93), albeit Martinez et al. found a decreased naïve CD4 population (93) rather than an increased CD44^hi^ CD62L^lo^ population in their transgenic mice. It has also been shown that a complete Treg repertoire with sufficient recognition power for autoreactive molecules is required for maintaining intestinal homeostasis (94). In the current study, SHP-1 is deleted at a distinct Treg developmental timeframe coinciding with the formation of the TCR repertoire. Although there was no prominent overall defect of positive and negative selection, Treg cells might have developed expressing an alternative TCR repertoires, which may be less diversified or specific for endogenous antigens, thereby less equipped for controlling autoantigen-driven T cell activation *in vivo*. This might manifest in increased levels of antigen-experienced Tcon cells, as we have observed in the *Foxp3* ^Cre+^ *Shp-1* ^f/f^ mice. Whether there is a critical developmental time point for SHP-1 expression to control the size of the CD44^hi^CD62L^lo^ population remains to be elucidated.

As a second independent explanation, the enlarged CD44^hi^ CD62L^lo^ population could also derive from an increase in ex-Treg cells. We therefore assessed Treg lineage stability *in vivo* using the tdTomato lineage tracing model. We found that SHP-1-deficient Treg cells are more likely to lose Foxp3 expression and become ex-Tregs, which is consistent with the potential fragile Treg paradigm, as indicated by increased AKT phosphorylation, suggesting a previously unappreciated new role for SHP-1 in Treg cells. We tested whether this “ex-Treg” population was due to a loss of IL-2-stabilized Foxp3 expression. *Foxp3* ^Cre+^ *Shp-1* ^f/f^ Treg cells showed levels of CD25 expression comparable to control Treg cells (**Supplementary Figure 2A**). Moreover, when stimulated with IL-2 *in vitro*, both *Foxp3* ^Cre+^ *Shp-1* ^f/f^ and *Foxp3* ^Cre+^ Treg cells maintain comparable level of Foxp3-expressing T cells (**Supplementary Figure 5C**). This data suggests SHP-1 does not affect the IL-2 signaling required to maintain Foxp3/Treg lineage. While ex-Treg cells might contribute to the increased antigen-experienced CD4 T cell population, ex-Treg cells alone cannot explain the expansion of all antigen-experienced T cells in the *Foxp3* ^Cre+^ *Shp-1* ^f/f^ mice, since there is also an increase in the percentage of CD44^hi^ CD62L^lo^ within the CD8 population.

Besides being antigen-experienced potentially auto-reactive T cells or ex-Treg, these CD44^hi^ CD62L^lo^ cells may belong to the “virtual memory” category. SHP-1 deficiency in Treg might promote the accumulation of these virtual memory T cells, in which some naive CD4 and CD8 T cells express CD44^hi^ CD62L^lo^ memory-like phenotype before encountering with their cognate antigen (95–97). These three possible explanations are not mutually exclusive and may all contribute to the increase in CD44^hi^ CD62L^lo^ CD4+ T cell.

In summary, our data reveal a previously unrecognized role of SHP-1 in mediating the function of Treg cells under physiological and patho-physiological conditions. Our data demonstrate that SHP-1 regulates AKT phosphorylation and Foxp3 stability in Treg cells. Moreover, our *in vivo* data suggest that SHP-1 is critical for optimal Treg-mediated suppression in an inflammatory environment. Since our findings are based on acute models of inflammation, it remains to be tested how SHP-1 is involved in the control of chronic inflammation. Interestingly, deficient SHP-1 expression has been associated with chronic inflammatory diseases such as psoriasis (98) and multiple sclerosis (99), which would potentially point towards a similar loss of function associated with SHP-1 insufficiency. Future studies are required to explore whether inducing SHP-1 expression in Treg cells would alleviate the diseases. On the contrary, functionally unstable and less suppressive Treg cells may be therapeutically preferrable in a tumor environment. Since loss of SHP-1 results in resistance to suppression in effector T cells (62) as well as functionality of Treg cells, SHP-1 inhibition may be a promising target for tumor clearance. While two phase I trials for malignant melanoma and advanced cancer malignancies using the SHP-1 inhibitor sodium stibogluconate (SSG) have failed due to the lack of efficacy on tumor burden while inducing strong toxic side-effects (100), retroviral SHP-1 knockdown together with immune checkpoint blockade has been effective in recruiting low affinity T cells for antitumor function (101). The resulting resurrection of SHP-1 as a potential drug target in the context of cancer emphasizes the need to gain a better functional understanding of the role of SHP-1 in various cellular subsets.

## Data availability statement

The raw data supporting the conclusions of this article will be made available by the authors, without undue reservation.

## Ethics statement

The animal study was reviewed and approved by The Institutional Animal Care and Use Committee at the University of Virginia.

## Author contributions

QG and UL conceived and designed the study and contributed to the data analyses and writing of the manuscript. QG performed the experiments, and KT analyzed and scored the histology slides in a blinded manner. All authors contributed to the article and approved the submitted version.
