# Supplementary material for: Treg-specific deletion of the phosphatase SHP-1 impairs control of inflammation in vivo
Source: Front Immunol. 2023 Mar 16;14:1139326. doi: 10.3389/fimmu.2023.1139326 (PMC10060847; doi:10.3389/fimmu.2023.1139326)
Supplement: Supplementary file 1 [file DataSheet_1.pdf]

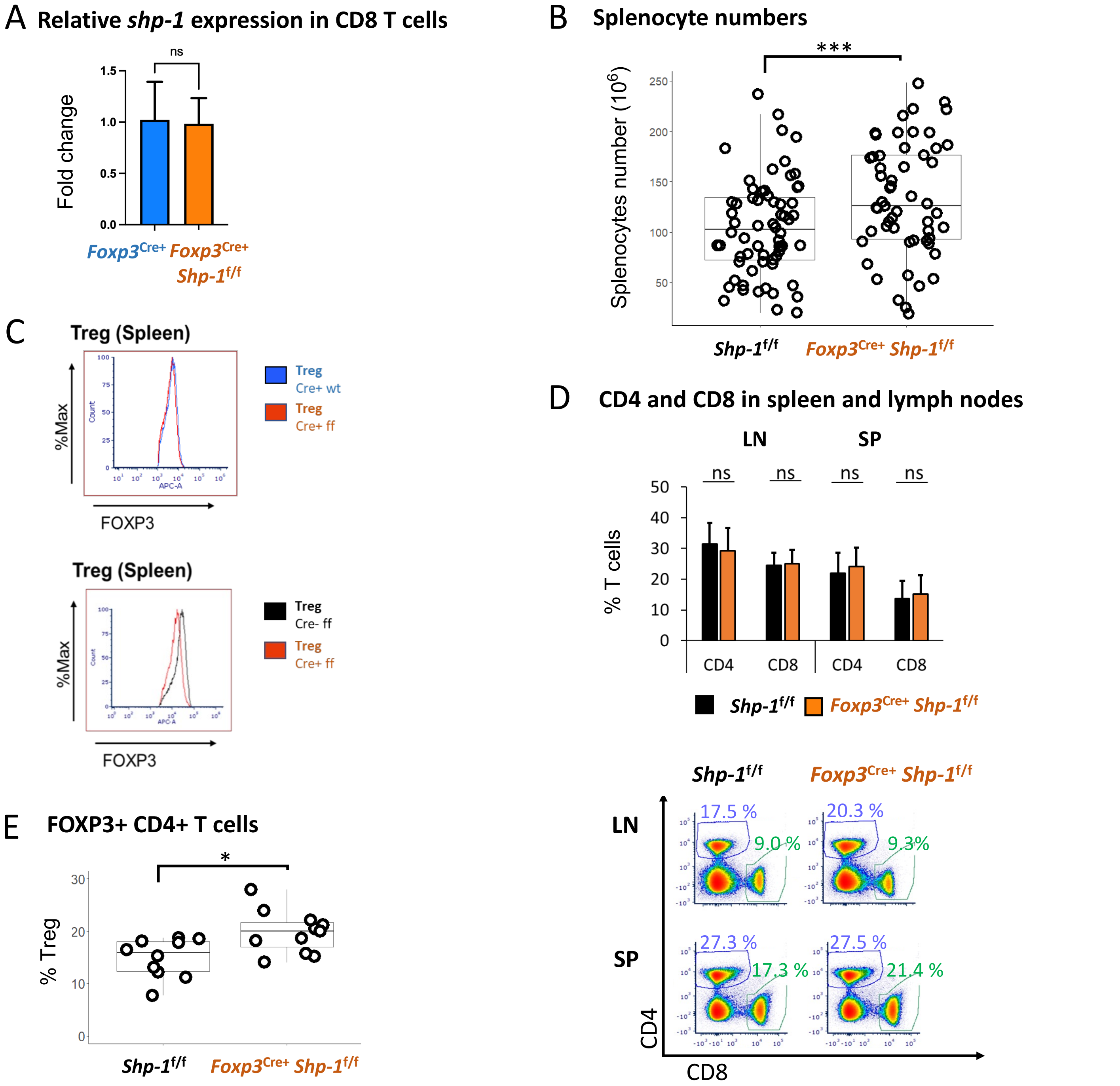

Supp. Figure 1 *Foxp3-Cre confers slight phenotypic changes*. (**A**) Relative *shp-1* mRNA expression levels in CD8+ T cells as measured by qPCR. (**B**) Total number of splenocytes isolated from 6-8 weeks old mice of indicated genotypes. Data are derived from 14 independent experiments. Each dot represents an animal; n=52 and 67, p-value= 0.0043. (**C**) Representative FOXP3 expression levels were assessed on live CD4+ FOXP3+ cells by flow cytometry. Data are gated: singlets → live cells → CD3 → CD4 → FOXP3 (**D**) Representative flow cytometry data with indicated percentages of CD4+ and CD8+ subpopulations. Average percentages of CD4+ and CD8+ T cells in lymph nodes and spleens of mice with indicated genotypes. Data are gated: singlets → live cells. Data points collected from 5 independent experiments with 8 mice/genotype. (**E**) Percentage of FOXP3+ Treg cells in total CD4+ T cells from cells. Data are gated: singlets → live cells → CD3 → CD4. Each dot represents an animal. p-value = 0.0102. n=11 for each genotype. ns = not significant.

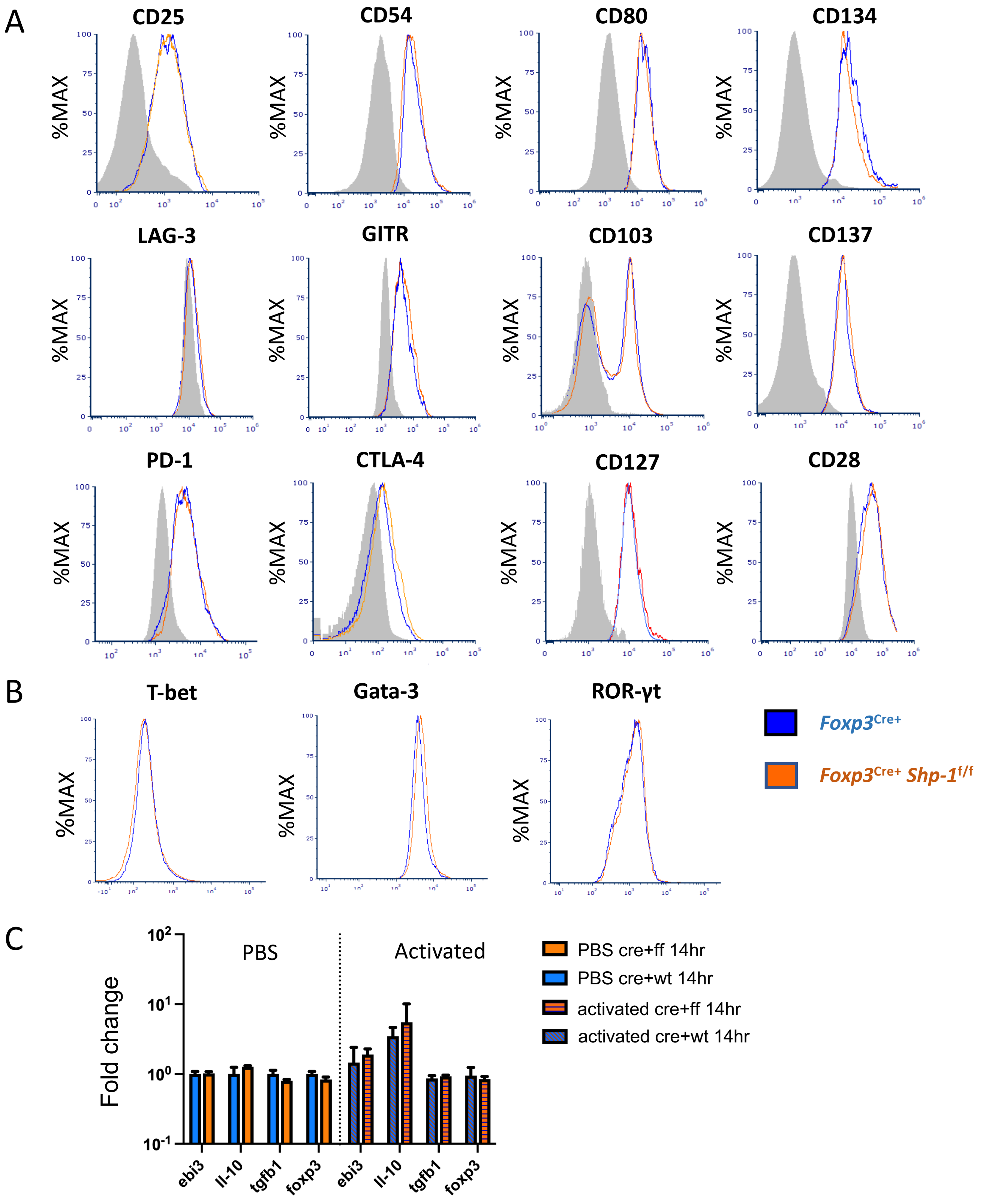

<u>Supp. Figure 2</u> *Phenotypic analyses of 6-8 weeks old Foxp3*$^{Cre+}$ Shp-1$^{f/f}$ *and Foxp3*$^{Cre+}$ *control mice.* Histograms depict representative (**A**) surface and (**B**) intracellular protein expression levels of live CD4+ FoxP3+ cells as assessed by flow cytometric analyses. Data are gated: singlets → live cells → CD3 → CD4 → FOXP3. (**C**) Relative cytokine expression of PBS treated or CD3/CD28 overnight stimulated splenic CD4+CD25+ Treg cells isolated from 6-8 weeks old mice of the indicated genotypes, n= 3 for each genotype treated with PBS, n= 5 for each genotype with overnight stimulation. Treg cells were incubated for 14-18 hours with plate-bound anti-CD3 (150 ng/ml) and soluble anti-CD28 (500 ng/ml). Error bars represent SD.

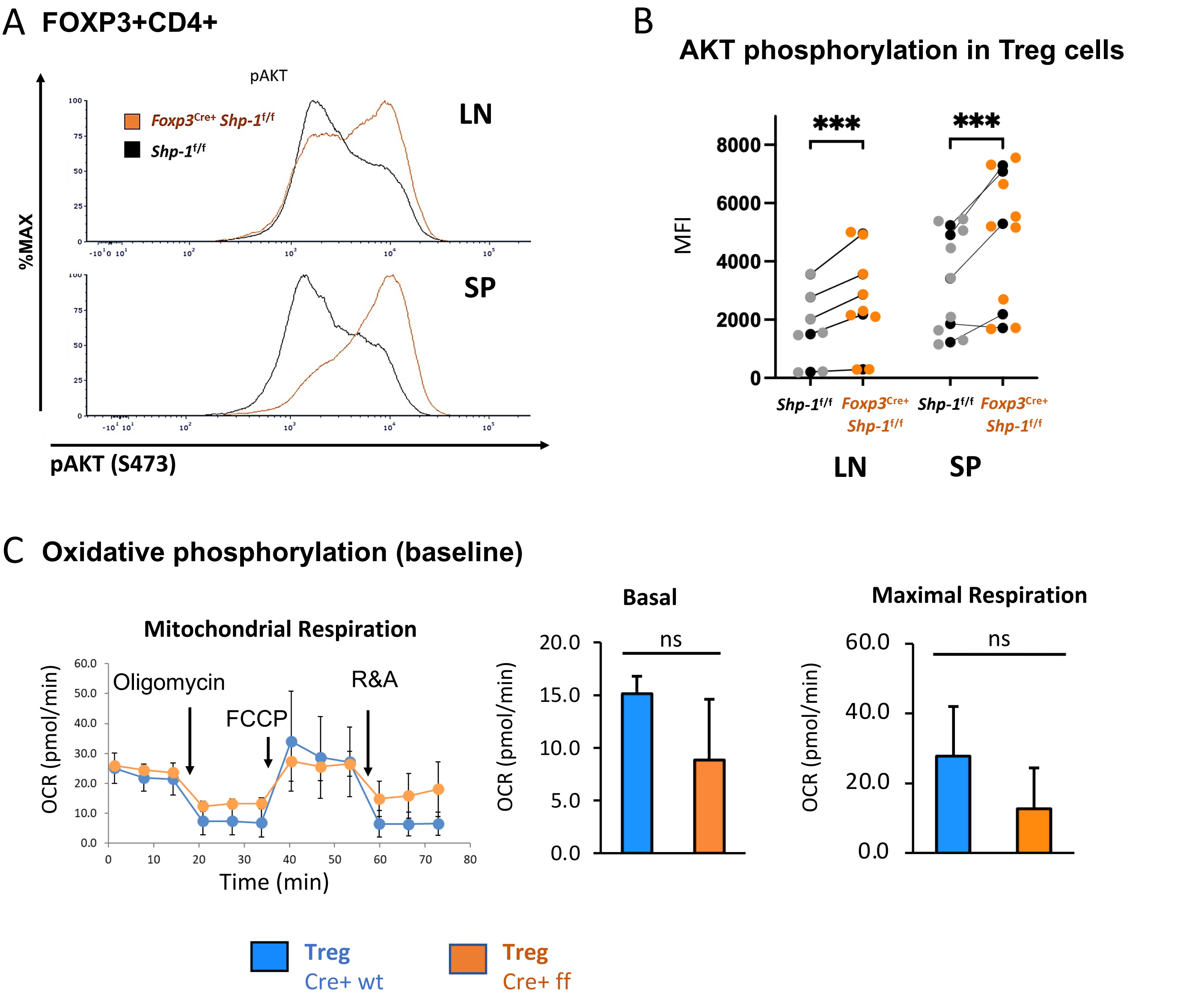

Supp. Figure 3 *Treg specific SHP-1 deletion increases phosphorylation of AKT and affects cellular metabolism.* (**A**) AKT phosphorylation (Ser473) in freshly isolated Treg of 6-8 weeks old Foxp3^{Cre+} Shp-1$^{f/f}$ or Shp-1$^{f/f}$ control mice. Data are representative of 4 independent experiments. (**B**) pAKT (Ser473) MFI of Foxp3^{Cre+} Shp-1$^{f/f}$ mutant and Shp-1$^{f/f}$ control Treg cells were measured. Colored dots represent each data point. Average value for each genotype in each experiment was indicated with black dots. Two-way ANOVA test was performed for statistics. P-values obtained for genotype effect was shown in figure. Data are gated singlets → live cells → CD3 → CD4 → FOXP3 (**C**) Basal and maximal mitochondrial respiration/OCR of freshly isolated CD4+CD25+ Treg cells of Foxp3^{Cre+} Shp-1$^{f/f}$ ("cre ff") or Foxp3^{Cre+} Shp-1$^{wt/wt}$ ("cre wt") control mice were measured using a Seahorse bioanalyzer. Error bar represents (**B**) SD and (**C**) s.e.m., unpaired t test, *$p < 0.05$, **$p < 0.01$, ***$p < 0.001$, ****$p < 0.0001$. ns = not significant.

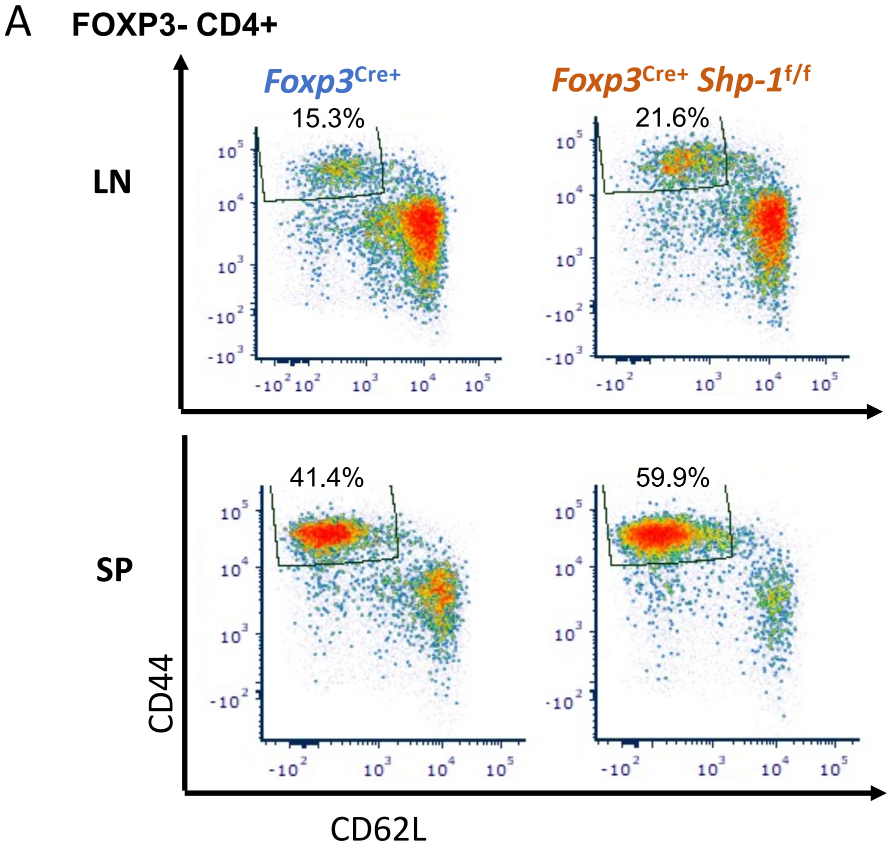

Supp. Figure 4 *Treg-specific SHP-1 deletion affects CD4+ Tcon cell population*. Percentages of CD44^{hi} CD62L^{lo} cells within CD4+ FOXP3- Tcon cells in the lymph nodes and spleen of *Foxp3*$^{Cre+}$ *Shp-1*$^{f/f}$ or *Foxp3*$^{Cre+}$ mice. Data are representative of 2 independent experiments. Gated: singlets → live → CD4 → FOXP3-.

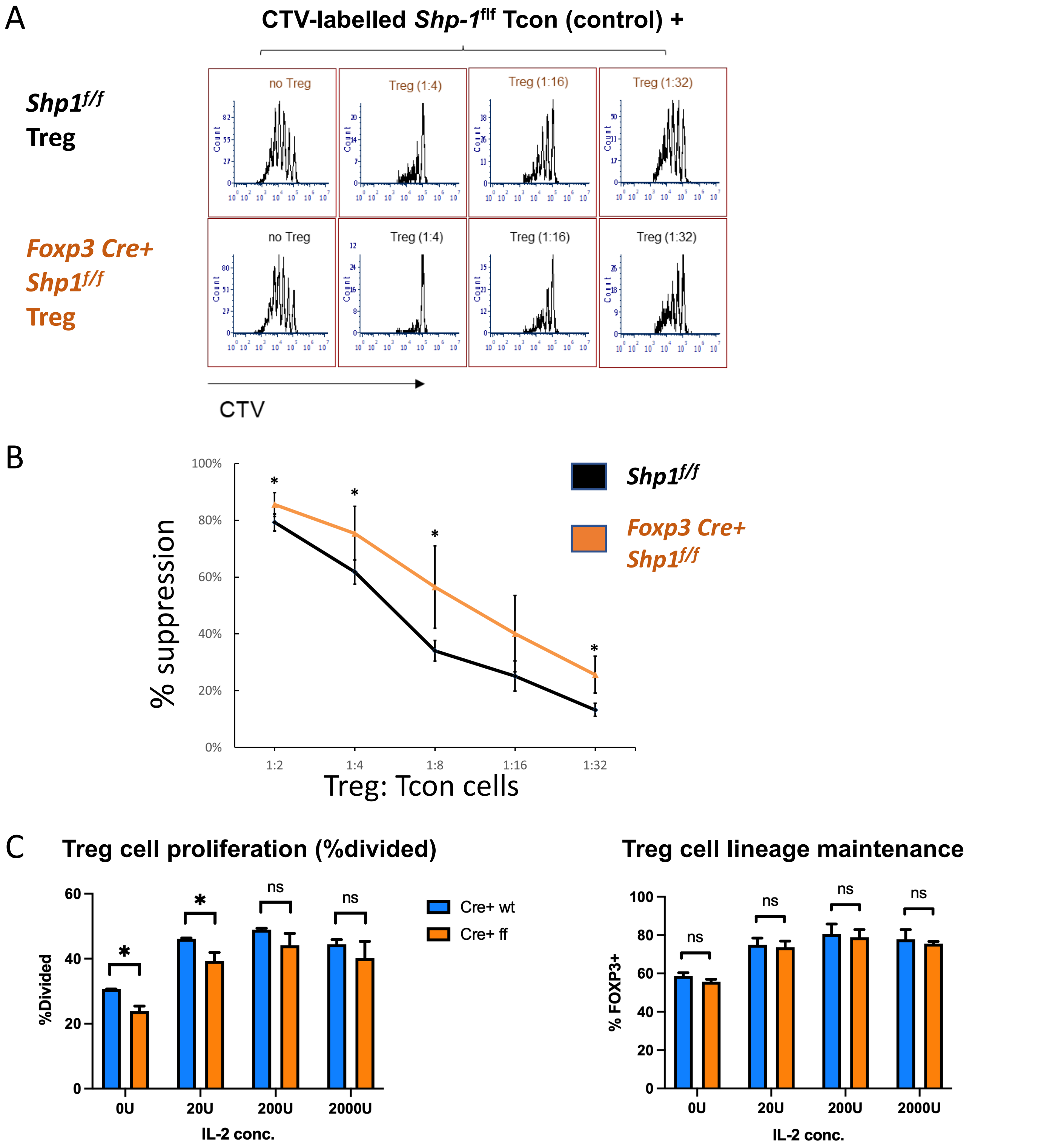

Supp. Figure 5 *SHP-1-deficient Treg cells display increased suppressive activity in vitro*. (**A** and **B**) Treg (CD4+CD25+ isolated from *Foxp3*$^{\text{Cre+}}$ *Shp-1*$^{\text{f/f}}$ mutant or *Shp-1*$^{\text{f/f}}$ control mice) and CTV-labeled Tcon (CD4+CD25- from control mice) cells were co-cultured at the indicated ratios. (**A**) Histogram depicts CTV dilution within Tcon cells as a measurement of proliferation. Data are representative of 5 independent experiments with n= 2 to 3 mice (6-9 weeks old) of each genotype for each experiment. (**B**) Suppression capacity of Treg cells based on data obtained in A. (**C**) (*Left*) Treg cell proliferation after 3 days of IL-2 stimulation (0 U, 20 U, 200 U, 2000 U). Treg cells were derived from *Foxp*3$^{\text{Cre+}}$ *Shp-1*$^{\text{f/f}}$ mutant or *Foxp3*$^{\text{Cre+}}$ control mice. (*Right*) Percentage of Treg cells that maintain Foxp3 expression following 3 days of IL-2 stimulation. n= 3 mice for each bar. Error bar represents SD. *, p≤ 0.05. ns = not significant.

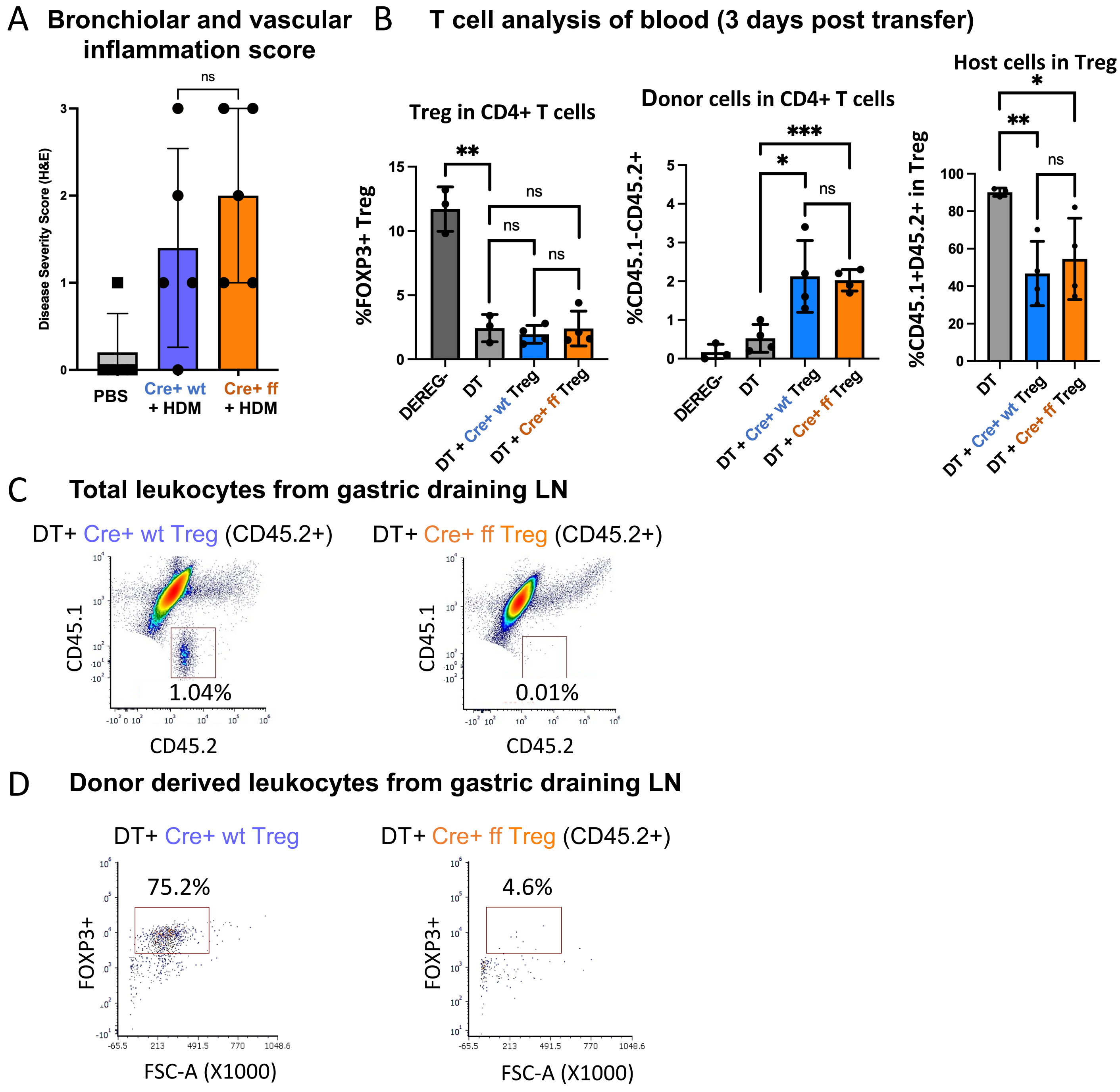

<u>Supp. Figure 6</u> *SHP-1 is required for the suppressive functionality of Treg cells in vivo.* (**A**) HDM-induced AAI disease severity score based on bronchiolar and vascular inflammation (H&E histology). (**B**) To assess the presence of total (left panel), donor-derived (CD45.1-CD45.2+) (middle panel), and host-derived (CD45.1+CD45.2+) Treg cells (right panel), blood was drawn at day 3 post transfer and analyzed by flow cytometry (note: % of donor cells (CD45.1- CD45.2+) in DEREG- and DT represents baseline autofluorescence). n = 3 (DEREG- mice), 4 (DT), 4 (cre+ wt control Treg), 4 (cre+ ff mutant Treg). (**C**) Representative flow cytometric data of CD45.2+ CD45.1- donor cells of indicated genotype 3 weeks post transfer. Host cells are CD45.2+ CD45.1+. Percentages indicate donor-derived T cells within gastric draining lymph nodes. Data are gated on singlets → live cells → CD45 (**D**) Representative flow cytometric data of FOXP3 expression within CD45.2+ CD45.1- donor cell population. Data are gated on singlets → live cells → CD45 → CD45.2+ CD45.1-. (**A**) One-way ANOVA test was applied followed by Fisher's LSD for comparison across multiple conditions. (**B**) Unpaired t test. *p <0.05, **p<0.01, ***p<0.001. Error bar represents SD. ns = not significant.
